# Near-infrared II plasmonic porous cubic nanoshells for in vivo noninvasive SERS visualization of sub-millimeter microtumors

**DOI:** 10.1038/s41467-022-32975-w

**Published:** 2022-09-06

**Authors:** Linhu Li, Renting Jiang, Beibei Shan, Yaxuan Lu, Chao Zheng, Ming Li

**Affiliations:** 1 School of Materials Science and Engineering, Central South University, Changsha, Hunan 410083 China; 2 Department of Breast Surgery, The Second Hospital, Cheeloo College of Medicine, Shandong University, Jinan, Shandong 250033 China

**Keywords:** Nanophotonics and plasmonics, Biomedical materials, Nanofabrication and nanopatterning, Cancer imaging

## Abstract

In vivo surface-enhanced Raman scattering (SERS) imaging allows non-invasive visualization of tumors for intraoperative guidance and clinical diagnostics. However, the in vivo utility of SERS is greatly hampered by the strong optical scattering and autofluorescence background of biological tissues and the lack of highly active plasmonic nanostructures. Herein, we report a class of porous nanostructures comprising a cubic AuAg alloy nanoshell and numerous nanopores. Such porous nanostructures exhibit excellent near-infrared II plasmonic properties tunable in a broad spectral range by varying the pore features while maintaining a small dimension. We demonstrate their exceptional near-infrared II SERS performance varying with the porous properties. Additionally, near-infrared II SERS probes created with porous cubic AuAg nanoshells are demonstrated with remarkable capability for in vivo visualization of sub-millimeter microtumors in a living mouse model. Our near-infrared II SERS probes hold great potentials for precise demarcation of tumor margins and identification of microscopic tumors.

## Abstract (web summary)

In vivo surface-enhanced Raman scattering (SERS) imaging allows non-invasive visualization of tumours for biomedical applications. Here, the authors report porous cubic AuAg alloy nanoshells exhibiting plasmonic properties and porosity-dependant SERS in the second window of the near-infrared for in vivo tumour detection.

## Introduction

In vivo optical imaging using surface-enhanced Raman scattering (SERS) probes has attracted considerable attentions for effective surgical operation and therapeutic monitoring of tumors in living subjects^1–3^. SERS imaging exploits unique vibrational spectroscopic fingerprints of Raman molecules massively amplified by plasmonic nanostructures for specific tumor identification^4,5^. It offers great advantages over in vivo fluorescence imaging in terms of ultrahigh sensitivity, high spectral specificity, powerful multiplexing capability, and improved photostability^4,6^. Therefore, SERS imaging could be an ideal optical technique for tumor margin delineation in vivo, especially for identification of microtumors and satellite tumor foci, which provides complementary information to those clinically available whole-body imaging modalities, for instance, computed tomography, magnetic resonance imaging, and positron emission tomography^7,8^. A SERS probe is typically created with conjugated Raman molecules decorated onto plasmonic Au or Ag substrates, sometimes with an additional protective outer layer (e.g., silica, polymers, proteins)^4,9^. The performance of SERS probes largely relies on the plasmonic substrate and Raman molecules. The local electromagnetic field enhancement at hot spots of plasmonic nanostructures could lead to a SERS enhancement of >10^10^ ^10–13^. The much better chemical stability and biocompatibility of Au over Ag make Au a preferrable choice for in vivo biomedical applications. To boost the SERS performance, considerable efforts have been devoted to creating Au nanostructures with sharp tips, nanogaps, and rough surface^14,15^. However, a major bottleneck of in vivo SERS biomedical applications is the plasmonic substrates for creation of SERS probes mostly with plasmon absorption in the visible-near-infrared I region (visible: 400–700 nm; NIR-I: 700–900 nm), in which only limited tissue penetration depth can be obtained due to the strong optical absorption, scattering and autofluorescence of biological tissues^16–20^.

Comparably, near-infrared II (NIR-II, 1000–1700 nm) SERS imaging could afford higher spatial resolution and higher detection sensitivity owing to the further reduction of tissue scattering and autofluorescence at NIR-II wavelengths^16,20,21^. In vivo SERS applications require plasmonic materials with small dimensions (typically < 100 nm) and high SERS enhancement. Shifting plasmon absorption into the NIR-II region is often realized by increasing the total dimension and surface roughness of anisotropic Au nanostructures such as nanorods, nanostars, nanoflowers, nanoshells (NSs), and hollow nanocages^22–26^. However, these strategies usually produce NIR-II plasmonic nanostructures with a size of >200 nm at least in one dimension, consequently leading to inefficient tumor accumulation. In addition, considering the 1/λ^4^-dependence of Raman signal on the laser wavelength, Raman intensity is intrinsically weak when the NIR-II (usually 1064 nm) laser is used^27^, so that the plasmonic substrate with a relatively large enhancement factor is of paramount importance for NIR-II SERS imaging of high brightness. Therefore, it is highly desirable to develop NIR-II plasmonic substrates with appropriate particulate size (50–100 nm) and large near-field enhancement for in vivo NIR-II SERS applications. Hollow and porous Au nanostructures show great promise as multifunctional platforms owing to their distinctive plasmonic properties tunable across the visible-near-infrared range^28,29^. These porous Au nanostructures present built-in hot spots of high density for large near-field enhancement, whose plasmon resonance wavelengths can be effectively tuned by varying the pore size and number, porosity, and composition while maintaining the constant total dimensions. Au nanocages with hollow interiors display the plasmon tunability up to 1200 nm^30^; however, the galvanic replacement reaction between the Ag nanocubes (NCs) as the sacrificial template and HAuCl_4_ usually leads to the etching of the sharp corners, compromising the SERS enhancement. The plasmon absorption of Au NSs with hollow interiors could be also pushed into the NIR region by tailoring the geometry, but currently limited to the NIR-I region^31^. In addition, the dealloying process has been utilized for the fabrication of porous Au nanostructures, but it only produced porous structures with plasmon absorption limited in the visible region^32–34^. Our recent work developed a class of Au@Au-Ag dot-in-cubic nanoframe structures with excellent NIR-II plasmonic properties, which exhibits remarkable spectral tunability by the edge length and wall pore size^35^. Inspired by this work, we speculate that manipulation of the pore size and number on a Au NS could be an effective way to develop NIR-II plasmonic materials with superior plasmonic properties for in vivo SERS imaging.

Herein, we report a type of porous cubic (pc)-AuAg NSs with hollow interiors, whose plasmonic properties can be effectively tuned over the NIR range through simply controlling the pore features in the NS. The pc-AuAg NSs are prepared by the galvanic replacement reaction and Au/Ag co-deposition on the Ag NCs as the sacrificial template, followed by subsequent etching with H_2_O_2_. The pore size and number can be controllably adjusted by the HAuCl_4_ amount added, thereby allowing the modulation of the plasmonic properties over a broad spectral range in the NIR-II biowindow. The pc-AuAg NSs display superior NIR-II plasmonic properties for in vivo SERS imaging owing to the high-density hot spots from the nanopores. We demonstrate non-invasive and highly accurate NIR-II SERS visualization of macro- and microtumors in living mice using the pc-AuAg NS-based SERS probes with the NIR-II excitation. Therefore, this study establishes a route to porous plasmonic nanostructures with high-density nanopores, tunable plasmonic properties, and superior NIR-II SERS activity for in vivo SERS biomedical applications.

## Results and discussion

### Synthesis and characterization of pc-AuAg NSs

**Fig. 1:**
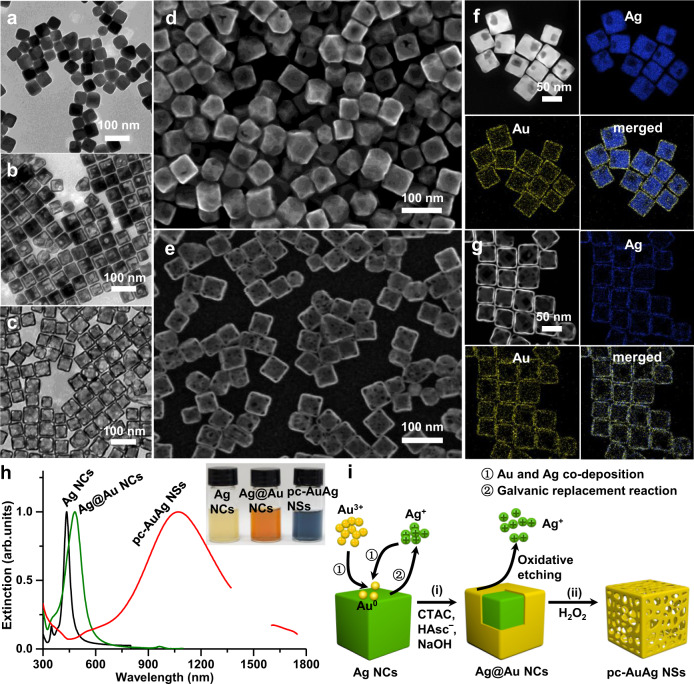
Structural and optical characterization of pc-AuAg NSs. **a**–**c** TEM images of **a** Ag NCs, **b** Ag@Au NCs, and **c** the corresponding pc-AuAg NSs after treatment with H_2_O_2_. **d**, **e** SEM images and **f**, **g** STEM images and the corresponding EDS elemental maps of Ag and Au of **d**, **f** Ag@Au NCs and **e**, **g** pc-AuAg NSs. **h** Optical extinction spectra of Ag NCs, Ag@Au NCs and pc-AuAg NSs (Inset is the optical photograph in aqueous solutions). **i** Schematic illustration of the synthetic process of pc-AuAg NSs, involving galvanic replacement reaction and Au/Ag co-deposition via reduction with HAsc¯, and treatment with H_2_O_2_. The synthesis of Ag@Au NCs in (**b**, **d**, **h**) was performed with 0.4 mL of 1.0 mM HAuCl_4_.

We first followed the published protocol to synthesize Ag NCs by a polyol route (Fig. 1a)^30^. Then, the conformal overgrowth of a Au shell on the Ag NC was performed with the rapid injection of HAuCl_4_ into an aqueous suspension of Ag NCs containing hexadecyltrimethylammonium chloride (CTAC), NaOH and L-ascorbic acid (H_2_Asc). The galvanic replacement reaction between Ag NCs and Au^3+^ takes place preferably at the corners and on the side faces of Ag NCs, leading to the etching of Ag atoms at these locations^35–37^. The subsequent reaction of Ag^+^ ions with OH¯ leads to the formation of Ag_2_O domains at the corners and partially on side faces, and the Au/Ag co-deposition via the reduction with HAsc¯ forms a conformal AuAg alloy overlayer, preventing the further etching of Ag atoms from the underlying Ag NCs. We can see from the transmission electron microscopy (TEM) and scanning electron microscopy (SEM) images in Fig. 1b the preservation of cubic morphology of Ag@Au NCs with truncated corners with the addition of 0.4 mL of 1.0 mM HAuCl_4_, the edge length of which increases to 55.6 (±4.3) nm from 51.0 (±3.8) nm of Ag NCs (Supplementary Fig. 1). In addition, both scanning transmission electron microscopy image (STEM) and energy-dispersive X-ray spectroscopy (EDS) elemental mapping clearly verify conformal coating of a thin Au nanoshell on each Ag NC (Fig. 1f). The satellite light contrast on the side faces of Ag@Au NCs evidently indicates the partial carving of Ag atoms from the side faces via the galvanic replacement reaction (Fig. 1b, d). As shown in Fig. 1d, the appearance of pores on the side faces also provides potent evidence for the carving of Ag atoms from the side faces. Finally, H_2_O_2_ was added to the suspension of Ag@Au NCs to etch away the Ag NC core and Ag_2_O domains of the nanoshell, forming the porous hollow nanoshells^36^. The TEM and SEM images obviously display the pores at the corners and on the side faces after the treatment with H_2_O_2_ (Fig. 1c, e). The EDS elemental mapping further supports the porous nanostructure of a AuAg alloy NS with hollow interior (Fig. 1g). The produced pc-AuAg NSs have a similar edge length to their corresponding Ag@Au NCs (Supplementary Fig. 1). The Au:Ag atomic ratio in the pc-AuAg NSs is 0.82 determined by inductively coupled plasma-optical emission spectroscopy (ICP-OES). The above results unambiguously demonstrate the successful synthesis of pc-AuAg NSs in this work. We must point out that the rapid injection of HAuCl_4_ is critical for the formation of surface pores because enough Au^3+^ ions are required to etch the Ag atoms from the corners and side faces of Ag NCs, accompanying with the formation of Ag_2_O domains that are eventually removed away for the nanoshell pore generation. Otherwise, the slow titration of HAuCl_4_ will produce pores exclusively at the corners rather than on the side faces, which was previously reported in the literature^37^.

The synthesis of the pc-AuAg NSs was also followed using UV-vis-NIR spectroscopy. The Ag NCs and Ag@Au NCs synthesized with 0.4 mL of 1.0 mM HAuCl_4_ exhibit strong plasmon bands at 434 nm and 481 nm, visually seen in light-yellow and bright orange colors, respectively (Fig. 1h). After reaction with H_2_O_2_, the plasmon band of the resulting pc-AuAg NSs undergoes a significant redshift to 1070 nm in the NIR-II region, appearing in a dark blue color in the aqueous suspension. The hollow structure and numerous pores of the nanoshell collectively contribute to the remarkable NIR-II plasmon absorption of pc-AuAg NSs. The synthetic philosophy of the pc-AuAg NSs is primarily based on the rapid galvanic replacement reaction and the simultaneous deposition of Au/Ag atoms using Ag NCs as the sacrificial template in the presence of CTAC, NaOH, and H_2_Asc (Fig. 1i). The etching of the internal Ag NC core and Ag_2_O domains produces porous AuAg NSs with nanopores at the corners and on the side faces, forming electromagnetic hot spots for NIR-II plasmon absorption.

**Fig. 2:**
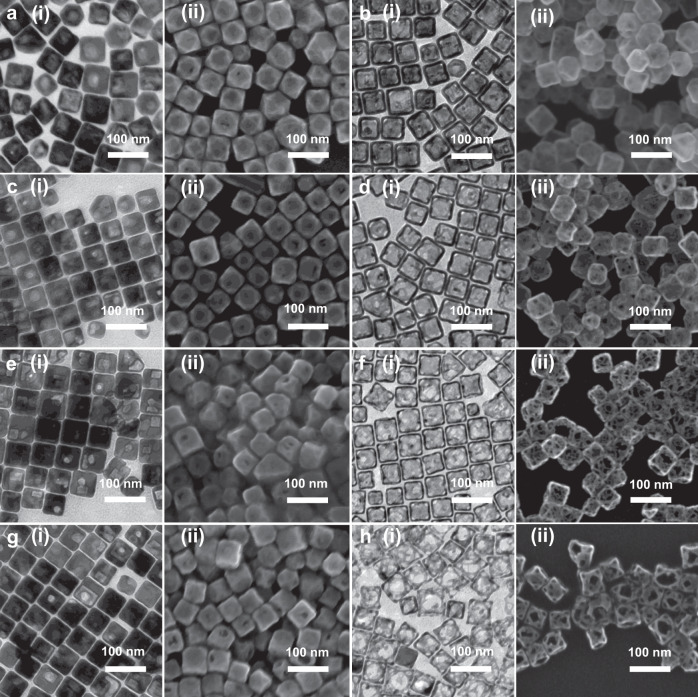
Tunable porous properties of pc-AuAg NSs. **a**–**h** TEM images (i) and SEM images (ii) of Ag@Au NCs and pc-AuAg NSs synthesized with **a**, **b** 1.2 mL, **c**, **d** 0.6 mL, **e**, **f** 0.3 mL, and **g**, **h** 0.1 mL 1.0 mM HAuCl_4_ (**a**, **c**, **e**, **g**) before and (**b**, **d**, **f**, **h**) after etching with H_2_O_2_. The dimensional parameters of all these samples are shown in Supplementary Figs. 1 and 2.

We argue that the HAuCl_4_ amount could be an effective parameter to tailor the porous properties of pc-AuAg NSs. Thus, we next examined the effects of the HAuCl_4_ amount on the synthesis of pc-AuAg NSs. Figure 2 shows the TEM and SEM images of the resulting structures synthesized with varied volumes of 1.0 mM HAuCl_4_ before and after etching with H_2_O_2_. We can see the cubic morphology with truncated corners for all these Ag@Au NCs synthesized with different volumes of HAuCl_4_. After etching with H_2_O_2_, fewer pores were generated in the pc-AuAg NSs synthesized at a volume of 1.2 mL HAuCl_4_; however, more pores in the pc-AuAg NSs show up with a decrease of the volume of HAuCl_4_. At a volume of 0.1 mL HAuCl_4_, large-sized pores can be seen in the resulting pc-AuAg NSs, which may originate from the merging of pores in close proximity to each other. These pc-AuAg NSs have a similar cubic morphology and edge length to their original Ag@Au NCs, except for hollow interior and additional pores in the nanoshell (Supplementary Figs. 2 and 3). Our rationale is that an increase in the HAuCl_4_ amount would form a thicker AuAg alloy overlayer completely covering on the Ag NC surface, and the AuAg alloy layer can survive the H_2_O_2_ etching process, so that fewer pores can be seen in the resulting pc-AuAg NSs. In contrast, a low amount of HAuCl_4_ only would lead to partial surface coverage of the Ag NCs with the AuAg layer, as a consequence forming more pores after H_2_O_2_ treatment. Therefore, the present approach enables the flexible tuning of pores of pc-AuAg NSs through simply varying the HAuCl_4_ amount.

### Optical properties and SERS performance of pc-AuAg NSs

**Fig. 3:**
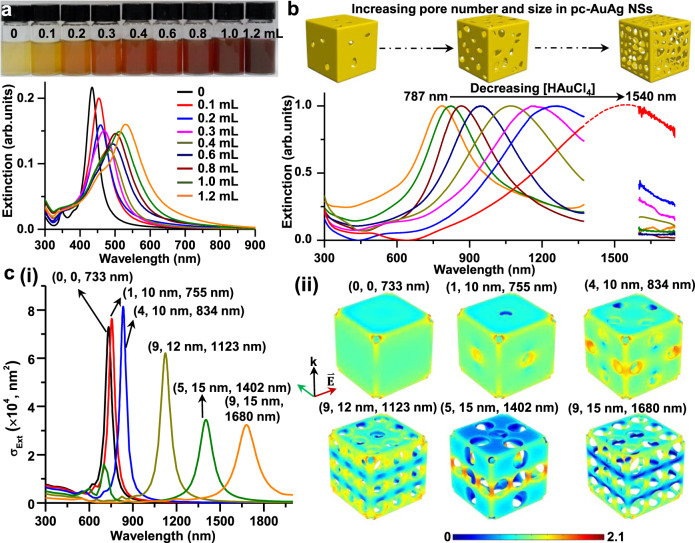
Tunable plasmonic properties of pc-AuAg NSs. **a** Extinction spectra and photographs of aqueous suspensions of Ag@Au NCs synthesized with different volumes of 1 mM HAuCl_4_. **b** Extinction spectra of the corresponding pc-AuAg NSs made with different volumes of 1 mM HAuCl_4_ (left to right: 1.2, 1.0, 0.8, 0.6, 0.4, 0.3, 0.2, and 0.1 mL). The structural evolution of pc-AuAg NSs with the increasing pore number is schematically shown. **c** (i) Calculated extinction cross-section (σ_Ext_) spectra and (ii) near-field (lg(|*E*|/|*E*_0_|)) distribution at the plasmon resonance wavelength (λ) of pc-AuAg NSs with representative pore number (N) and diameter (d) denoted as (N, d, λ).

We next monitored the evolution of the optical properties of Ag@Au NCs and pc-AuAg NSs with the addition of various amounts of HAuCl_4_. As shown in Fig. 3a, the plasmon resonance wavelength of Ag@Au NCs is gradually redshifted to 530 nm from 434 nm of Ag NCs with the volume of 1.0 mM HAuCl_4_ increasing from 0 to 1.2 mL. In addition, the intensity of the plasmon band shows an initial decrease and then an increase with a continuous addition of HAuCl_4_. The change of the plasmon band of Ag@Au NCs could be attributed to the deposition of a thin Au overlayer on the Ag NC surface, which yields pure Au NC-like plasmon features. The initial decrease in the plasmon intensity may be due to the Ag dissolution from Ag NCs and the relatively weaker plasmon activity of the deposited Au layer compared with Ag, and the subsequent increase in the plasmon intensity could be attributed to the co-deposition of Au/Ag atoms via reduction with HAsc¯. After H_2_O_2_ etching, pc-AuAg NSs were formed through the removal of the Ag NC core and Ag_2_O domains, and the plasmon resonance wavelength progressively red-shifts from 787 to 1540 nm as the volume of 1.0 mM HAuCl_4_ decreases from 1.2 to 0.1 mL (Fig. 3b). We conclude that an increase in the pore size and number causes the red-shifting of the plasmon band of pc-AuAg NSs. Thus, the addition of few amounts of HAuCl_4_ could produce pc-AuAg NSs with NIR-II plasmonic properties.

To better understand the plasmonic properties of pc-AuAg NSs, the numerical finite-element analysis was conducted based on the COMSOL Multiphysics software to calculate the optical extinction spectra and near-field distribution of pc-AuAg NSs with varied pore sizes and number of the nanoshell, as depicted in Fig. 3c and Supplementary Fig. 4. For simplification, the geometric model was created with a pure Au NS of an edge length of 56 nm that has hollow interior and slightly truncated corners. The pc-Au NSs display pore size- and number-dependent plasmonic properties in terms of intensity and plasmon resonance wavelength. The plasmon resonance wavelength of the pc-Au NSs can be easily tuned into the NIR-II region upon increasing the pore size and number. It is clearly seen that the local near-field at the NIR-II resonance wavelength is significantly intensified in the pores of the pc-Au NSs, yielding high-density hot spots for electromagnetic enhancement of SERS. The above results demonstrate that pc-AuAg NSs display excellent plasmonic properties in the NIR-II region tunable through simply varying the pore features while maintaining a small total size suitable for in vivo biomedical applications.

**Fig. 4:**
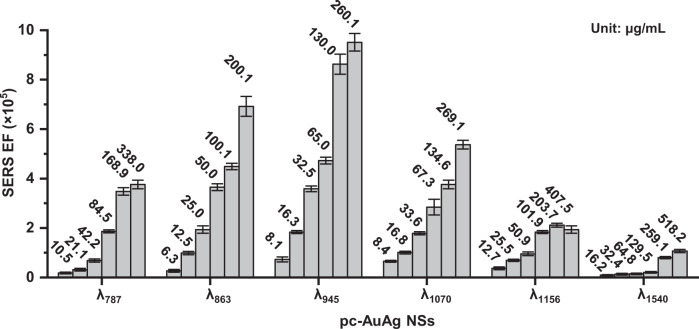
SERS performance evaluation. SERS enhancement factors of pc-AuAg NSs with various plasmon resonance wavelengths were measured at varied concentrations (unit: μg/mL). The concentration for each measurement of SERS enhancement factor is shown on top of each column for each type of pc-AuAg NSs, and IR-1061 dye was used as the Raman molecule at a fixed concentration of 1.0 μM in all these measurements. Data are presented as mean values ± SD (*n* = 37 independent experiments).

Figure 4 shows the NIR-II SERS performance of pc-AuAg NSs of various plasmon resonance wavelengths. The SERS measurements were carried out at varied concentrations of pc-AuAg NSs with the resonant Raman reporter IR-1061 dye and the non-resonant Raman reporter p-nitrothiophenol (p-NTP), respectively. The SERS intensity of IR-1061 dye-encoded pc-AuAg NSs under the NIR-II (1064 nm) laser is significantly higher than the non-resonant p-NTP-encoded pc-AuAg NSs under either 1064 or 785 nm laser (Supplementary Fig. 5). The analytical NIR-II SERS enhancement factor (EF) was calculated for quantitative comparison of SERS performance of these samples^27,38,39^. With IR-1061 dye as the resonant Raman reporter, the estimated NIR-II SERS EF of these pc-AuAg NSs exhibits a concentration dependence typically in a range of ~0.1–9.5 × 10^5^; the NIR-II SERS EF first increases and then decreases with the increasing plasmon resonance wavelength. The pc-AuAg NSs with the 945 nm plasmon resonance wavelength (denoted as pc-AuAg NSs-945 nm) have the highest NIR-II SERS EF, while the lowest SERS EF for pc-AuAg NSs with the 1540 nm plasmon resonance wavelength. The maximum NIR-II SERS enhancement takes place at a plasmon resonance wavelength blue-shifting from the laser excitation wavelength (1064 nm) used for the present SERS measurement, which is attributed to the antagonistic interplay between optical extinction and near-field enhancement in SERS enhancement, consistent with our previous reports^40,41^. Then, the limit of detection (LOD) of NIR-II SERS detection was estimated using pc-AuAg NSs-945 nm (Supplementary Fig. 6). Agarose phantoms were made with pc-AuAg NSs-945 nm of various concentrations and 1.0 μM IR-1061 dye, which were subjected to the NIR-II SERS measurement. Elevated brightness of the SERS images of these agarose phantoms was observed as the concentration of pc-AuAg NSs-945 nm increases. The intensity of the 1557 cm^−1^ SERS band is linearly proportional to the concentration of pc-AuAg NSs-945 nm, with a linear regression equation: *y* = 59.0*x* − 45.1 (*R*^2^ = 0.988). The LOD is determined to be 2.14 μg/mL, computed according to the IUPAC definition of LOD = 3σ/s, where σ and s represent the standard deviation of the blank measurement (*n* = 42) and the slope of the linear regression equation obtained above, respectively^42^. Thus, all these experimental data verify the exceptional NIR-II SERS activity of pc-AuAg NSs tunable by changing the pore size and number of the nanoshell, presenting great potential for in vivo SERS bioimaging.

### NIR-II SERS imaging of multicellular tumor spheroids

**Fig. 5:**
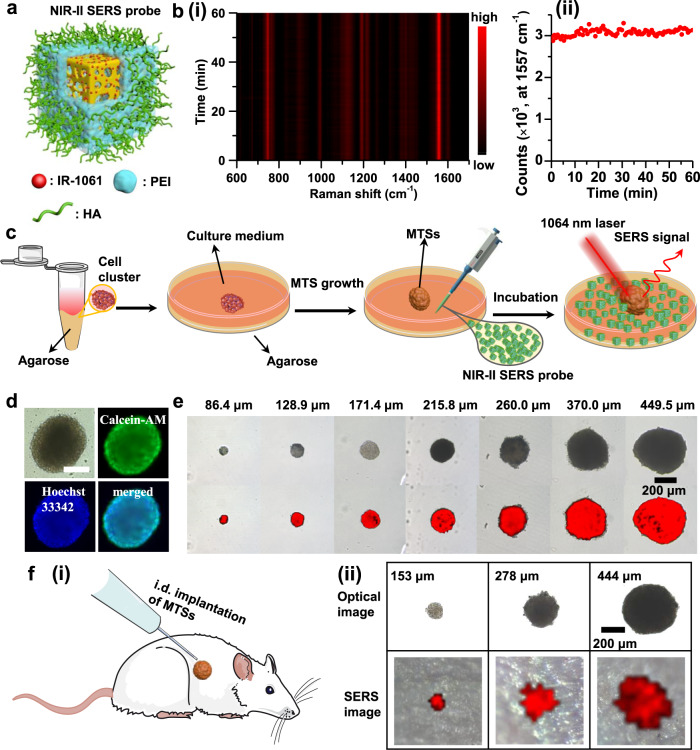
NIR-II SERS imaging of MTSs. **a** A schematic of a NIR-II SERS probe comprising a pc-AuAg NS core, IR-1061 dye as the Raman reporter, a PEI coating layer, and HA capping ligand. **b** (i) Time-dependent SERS spectra and (ii) the intensity change of the 1557 cm^−1^ SERS peak of the present NIR-II SERS probes over a 60 min continuous exposure to a 1064 nm-laser at a power of 80.1 mW (integration time: 1 s, objective lens: ×50). **c** Schematic illustration of the preparation process of MTSs and their in vitro NIR-II SERS measurements. **d** Optical photographs and fluorescence images of a representative MTS co-stained with Calcein-AM (green) and Hoechst 33342 (blue), confirming the live cells in the MTS. Scale bar: 200 μm. **e** Optical photographs and the corresponding NIR-II SERS images of MTSs of various sizes, created with the DCLS algorithm. **f** (i) Schematic illustration of the i.d. implantation of MTSs into a living mouse and (ii) the corresponding in vivo NIR-II SERS imaging of implanted MTSs of three different sizes (153, 278, and 444 μm in diameter). The 4T1 MTSs were first incubated with NIR-II SERS probes for 12 h and then washed with fresh cell culture medium, followed by implantation into a living mouse for SERS measurements (×5 objective lens, 7.4 mW laser power, 1 s integration time, 50 μm step size).

We next explored the potential of pc-AuAg NSs for in vivo NIR-II SERS imaging of microtumors. In the following experiments, the pc-AuAg NSs-945 nm were used to construct the NIR-II SERS probe because of the superior NIR-II SERS activity. The as-synthesized pc-AuAg NSs were first treated in an aqueous solution of 0.15 wt% poly(sodium 4-styrenesulfonate) (PSS) to replace the original surface CTAC, yielding PSS-modified pc-AuAg NSs charged negatively (Supplementary Figs. 7 and 8). As seen in Supplementary Fig. 9, Fourier-transform infrared spectra show the disappearance of -C-N- stretching bands at 920–950 cm^−1^ and the appearance of the -SO_3_- bands at 1010 and 1040 cm^−1^, confirming the complete replacement of CTAC with PSS on the surface of pc-AuAg NSs^43,44^. A NIR-II SERS probe was made with IR-1061 dye-encoded pc-AuAg NSs-945 nm encapsulated with a polyethyleneimine (PEI) outer layer and subsequently modified with hyaluronic acid (HA) endowing the active targeting ability toward CD44 receptor on the cell membrane of 4T1 cells^45^, as schematically illustrated in Fig. 5a and Supplementary Fig. 7. After polyethyleneimine coating, the zeta potential shifts to +19.6 (±0.4) mV from −19.7 (±0.38) mV of IR-1061 dye encoded porous NSs, and the further modification with HA renders the NIR-II SERS probes with negative zeta potential of −12.5 (±0.9) mV (Supplementary Fig. 8). This negative surface charge endows the present NIR-II SERS probe with resistance to non-specific protein adsorption and prolonging body systemic circulation^46^. Significantly, we observed negligible degradation of SERS signal of the NIR-II SERS probe over a 60 min continuous exposure to the 1064 nm laser at a power of 80.1 mW, indicating the superior photostability (Fig. 5b). We then assessed the in vitro cytotoxicity using the cell counting kit-8 (CCK-8) assay against 4T1 mouse breast cancer cells and L-02 human normal liver cells, respectively. The results show that both 4T1 and L-02 cells maintain cell viabilities of >89% even at a concentration of NIR-II SERS probes up to 200 μg/mL, indicating excellent in vitro biocompatibility (Supplementary Fig. 10).

Subsequently, we mimicked microtumors using multicellular tumor spheroids (MTSs) made through culturing cell clusters in cell culture medium, as illustrated in Fig. 5c^47,48^. Calcein-AM/propidium iodide/Hoechst 33342 co-stained experiments confirm the live cells in the MTSs with negligible dead cells observed even at the center of the MTS (Fig. 5d and Supplementary Fig. 11). After incubation with NIR-II SERS probes (50 μg/mL), these MTSs were subjected to the NIR-II SERS imaging in vitro. All these MTSs exhibit high SERS intensity over the entire tumor area, and the contour of the NIR-II SERS images can perfectly follow the shape of the MTSs (Fig. 5e and Supplementary Fig. 12). We also conducted the control experiment using IR-1061-encoded pc-AuAg NSs modified with poly(ethylene glycol) monomethyl ether thiol (mPEG-SH) (mPEG-modified NIR-II SERS probes). Only weak SERS signal was seen in the MTS after incubation with mPEG-modified NIR-II SERS probes, indicating the specific uptake of our present NIR-II SERS probes by 4T1 cells (Supplementary Fig. 13). Then, we performed in vivo SERS imaging using our NIR-II SERS probes, in which three MTSs of different sizes (153, 278, and 444 μm) were intradermally (i.d.) implanted at a 2 mm depth beneath the skin of a living mouse. Figure 5f clearly verifies the successful NIR-II SERS imaging of the three MTSs in the living mouse. Further, we observe strong background (e.g., optical scattering, autofluorescence) signal from the skin and its intrinsic Raman bands in a living mouse with 785 nm laser excitation (Supplementary Fig. 14), and verify that the combination of pc-AuAg NSs and IR-1061 dye possesses superior SERS performance under 1064 nm laser than those conventional plasmonic materials such as Au nanorods under either 785 or 1064 nm laser (Supplementary Fig. 15).

### In vivo NIR-II SERS imaging of tumors in a mouse model

**Fig. 6:**
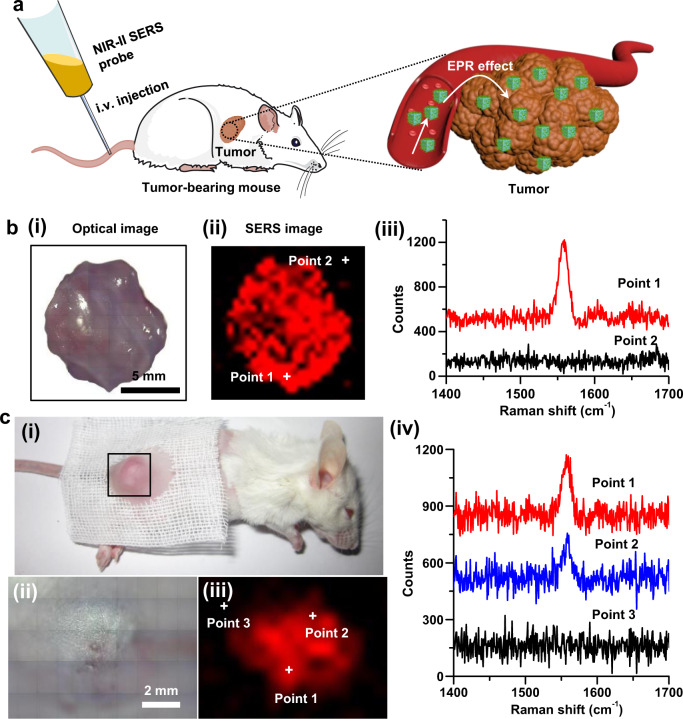
NIR-II SERS imaging of a solid tumor in a living tumor-bearing mouse. **a** Schematic representation of NIR-II SERS probes for in vivo SERS imaging of tumors. NIR-II SERS probes were intravenously administrated into a 4T1 tumor-bearing mouse, and thereby effectively accumulated in the tumor. **b** (i) Optical photograph, (ii) ex vivo NIR-II SERS image and (iii) representative SERS spectra at points 1 and 2 within and outside a tumor excised from a tumor-bearing mouse at 12 h post-administration of NIR-II SERS probes. **c** (i) Optical photograph of a 4T1 tumor-bearing mouse intravenously administrated with NIR-II SERS probes, (ii) zoom-in optical photograph of the tumor for in vivo NIR-II SERS imaging and (iii) the corresponding NIR-II SERS image of the tumor site directly taken from the mouse at 12 h post-administration, and (iv) representative SERS spectra at points 1, 2, and 3 as indicated in the SERS image (iii).

It has been demonstrated that our NIR-II SERS probes display excellent performance for in vivo SERS imaging of microtumors. Thus, we further studied in vivo SERS imaging of tumors in a tumor-bearing mouse model. As shown in Fig. 6a, the 4T1 tumor-bearing mouse model was established through inoculating 4T1 tumor cells (4 × 10^6^ cells) into the right sub-dermal dorsal area of the female mice with the tumor growth to approximate 150 mm^3^, followed by intravenous administration of NIR-II SERS probes (*n* = 3). Ex vivo NIR-II SERS imaging was first conducted on the tumor excised from the tumor-bearing mouse at 12 h post-administration. We can see from Fig. 6b that the resected tumor exhibits intense yet quite uniform SERS signal over the entire tumor, and the SERS image can depict clear demarcation of the resected tumor. Significant CD44 overexpression on the cell membrane of 4T1 breast cancer cells has been previously confirmed and extensively employed for the development of targeting drug delivery systems^45,49–51^. The effective tumor accumulation of NIR-II SERS probes may be attributed to the combination of the passive enhanced permeability and retention (EPR) effect and the active targeting of CD44 overexpressed on the 4T1 tumor cells^1,45,51,52^. We further examined the suitability of our NIR-II SERS probes for in vivo NIR-II SERS imaging of a tumor in a 4T1 tumor-bearing mouse. Figure 6c presents the SERS measurement of the tumor directly in a tumor-bearing mouse. The in vivo SERS image detects strong SERS signal at the tumor sites (points 1 and 2) while negligible outside the tumor (point 3). Moreover, our results showed that the in vivo NIR-II SERS image could delineate the boundary between the tumor and the surrounding healthy tissue. The time-dependent retention of the NIR-II SERS probes in the tumor was further investigated by inductively coupled plasma-mass spectrometry. It can be seen that the content of the NIR-II SERS probes in the tumor declines over time (Supplementary Fig. 16), suggesting that the NIR-II SERS imaging of the tumor should be done at the first two days after intravenous administration. Further, hematology and serum biochemistry experiments were carried out to examine whether our NIR-II SERS probes have significant systemic toxicity. Healthy mice were intravenously administrated with NIR-II SERS probes or PBS as the control, and the blood was collected at 24 h post-administration. As shown in Supplementary Figs. 17 and 18, no significant change is observed for all these blood biochemical indices. In addition, histological analysis of major organs (heart, liver, lung, spleen, and kidney) shows no obvious pathological changes and necrosis in 4T1 tumor-bearing mice after treatment with NIR-II SERS probes (Supplementary Fig. 19). The experimental results confirm that the present NIR-II SERS probes exhibit excellent biosafety with no obvious adverse effects on the liver/kidney functions and the immune response. The results clearly show that the pc-AuAg NSs are highly promising for in vivo SERS application with excellent plasmonic properties and SERS performance both in the NIR-II biowindow.

In summary, this work has successfully developed a class of NIR-II plasmonic porous nanostructures comprising a cubic AuAg alloy NS and high-density nanopores. The effective combination of the galvanic replacement reaction, Au/Ag co-deposition simultaneously at the corners and side faces of the Ag NC sacrificial template, and the subsequent H_2_O_2_ etching results in the formation of pc-AuAg NSs. The developed pc-AuAg NSs exhibit excellent NIR-II plasmonic properties tunable in a broad spectral range through simply varying the porous properties (i.e., size, number) while maintaining a small total size. We experimentally and theoretically demonstrated the porous property-dependent NIR-II SERS performance. We simplified the geometric model with fixed edge length, circular pore, and pure Au composition for numerical simulations of extinction spectra and near-field distribution through varying the pore diameter and number. In practice, pc-AuAg NSs synthesized experimentally have AuAg alloy composition with varied geometric shape, pore size, and pore shape. Although the simplification of geometric model could affect the position of plasmon resonance wavelength and the plasmon strength, it would not change the trend over pore size and number. Subsequently, we created the NIR-II SERS probe using the pc-AuAg NSs with optimal plasmonic properties. It is demonstrated that our NIR-II SERS probes exhibit remarkable capability for in vivo SERS visualization of microtumors in animals. This NIR-II SERS probe could also delineate the tumor margin directly in a living mouse model following the intravenous administration. The non-invasive SERS imaging capability of our NIR-II SERS probes in living animals opens up an opportunity to intraoperative guidance of complete tumor resection and longitudinal monitoring of cancer therapeutic response^6–8,53^. We find that the tumor accumulation of NIR-II SERS probes declines over prolonging time following intravenous administration, so that longitudinal SERS imaging should be performed prior to metabolic elimination of NIR-II SERS probes from the tumors. The further modification of NIR-II SERS probes with functional biological substances such as cell membrane and exosomes could improve the tumor accumulation and prolong the tumor retention for dynamic monitoring of a tumor in a living subject for treatment assessment^54,55^. We also envision that the pc-AuAg NSs could offer a potential multifunctional platform, e.g., NIR-II photothermal agents, drug carriers, and multi-modal imaging contrast agents, for cancer theranostics^35,56^.

## Methods

### Animals and cell lines

Female Balb/c mice (four weeks, 13–16 g) were ordered from Changzhou Cavens Laboratory Animal Co. Ltd. This research complies with all relevant ethical regulations. All procedures of animal experiments were approved by the Institutional Animal Care and Use Committee at Central South University (Protocol No. 2020sydw0724).

Both 4T1 mouse breast cancer cells and L-02 human normal liver cells were obtained from American Type Culture Collection (ATCC) and cultured at 37 °C in a 5% CO_2_ incubator in culture medium that is composed of 10% fetal bovine serum (FBS) and 1% penicillin-streptomycin in DMEM.

### Synthesis of pc-AuAg NSs

First, Ag nanocubes (NCs) were synthesized according to a polyol process previously developed by Xia’s group^30^. Then, pc-AuAg NSs were synthesized with Ag NCs as the sacrificial template. In a typical protocol, 1 mL of 100 mM L-ascorbic acid (H_2_Asc) aqueous solution was injected into 4 mL of 100 mM CTAC aqueous solution together with the addition of 1 mL of 200 mM NaOH aqueous solution under vigorously magnetic stirring. Afterward, 0.3 mL of aqueous solution of as-synthesized Ag NCs with the concentration of 0.74 mg/mL was added into the reaction mixture, immediately followed by the rapid injection of various volumes of 1.0 mM HAuCl_4_ aqueous solution. The reaction continued for 10 min, followed by the dropwise addition of 500 µL of H_2_O_2_ (3.0 vol%). The reaction mixture was kept still overnight at room temperature. The resulting products were purified by centrifugation at 4500 × *g* for 15 min and washing with ultrapure water. The pellets were pc-AuAg NSs with various porous structures, depending on the amount of HAuCl_4_ added. These pc-AuAg NSs were finally re-dispersed in ultrapure water for the following use, whose concentrations were determined by ICP-OES.

### Fabrication of NIR-II SERS probes

To synthesize the NIR-II SERS probes, the as-synthesized pc-AuAg NSs were centrifuged at 5900 × *g* for 15 min, followed by incubation for 2 h in 0.15 wt% PSS. The reaction mixture was centrifuged at 5900 × *g* for another 15 min, and the precipitate was re-dispersed in 0.15 wt% PSS. The above process was repeated three times to produce PSS-modified pc-AuAg NSs. 10 mL of aqueous suspension of PSS-modified pc-AuAg NSs was then added with 1 μM IR-1061 dye as the resonant Raman reporter. The reaction mixture was subsequently incubated for 6 h, and then purified successively by centrifugation at 5900 × *g* for 15 min and washing with ultrapure water. The precipitate was re-dispersed into 10 mL ultrapure water, and then added with 100 μL of 1 mg/mL PEI under magnetic stirring. After 15 min incubation, the reaction mixture was centrifuged at 5900 × *g* and re-dispersed in 10 mL of ultrapure water, followed by the addition of 100 μL of 10 mg/mL HA. The reaction continued for 4 h, and the reaction mixture was then centrifuged at 5900 × *g* for 15 min. The resultant precipitate was IR-1061 encoded pc-AuAg NSs encapsulated in a PEI overlayer with HA modification (called NIR-II SERS probes hereafter), which were dispersed in ultrapure water for storage at 4 °C.

### Numerical simulations of plasmonic properties

Calculations of optical extinction spectra and three-dimensional (3D) near-field distribution were performed by finite element method using a commercial software package COMSOL Multiphysics 5.5. To simplify the calculation, the geometric model of the pc-AuAg NS was created with a pure Au NS with hollow interior and truncated corners. The calculated domain was delimited by the perfectly matched layers. The dimensional parameters (i.e., edge length, shell thickness) were set according to the statistical results from the TEM image. The dielectric properties of Au were taken from the data of Johnson and Chirsty^57^. The surrounding medium was water with a refractive index of 1.33. Optical extinction spectra were calculated in the wavelength range of 300–2000 nm, and the mesh size in the 3D simulation space was set to be 2.8–38.5 nm with finer levels in the nanostructure and surrounding medium domains. The plane wave excitation source was polarized along the z-axis, and propagated along the x axis with the magnitude of electric field of 1 V/m.

### In vitro cytotoxicity

Cell counting kit-8 (CCK-8) viability assay was performed to evaluate the cytotoxicity of NIR-II SERS probes against 4T1 and L-02 cells, respectively. Typically, cells were seeded in 96-well plates at a density of 8 × 10^3^ cells/well (*n* = 3) and after 24 h incubation the culture medium was replaced with fresh culture medium containing NIR-II SERS probes of various concentrations (6.25, 12.5, 25, 50, 100, and 200 μg/mL). Control experiments were performed without NIR-II SERS probes as well. After incubation for another 24 h, the cells were washed with PBS (pH 7.4) and then cell viability was examined by CCK-8 assay. Specifically, 100 µL of DMEM was added to each well followed by the addition of 10 µL of CCK-8 solution. The absorbance at 450 nm of each well was measured on a Tecan Spark Microplate Reader with the background absorbance of culture medium subtracted.

### Growth of multicellular tumor spheroids

The multicellular tumor spheroids (MTSs) of various sizes were made using 4T1 cells according to the well-established protocol previously reported in the literature^47,48^. First, 5 μL of 2.0 wt% agarose-containing DMEM medium pre-sterilized at 120 °C was added to a microcentrifuge tube. Once the agarose-containing DMEM medium was solidified, 100 μL of 4T1 cell-containing culture medium at varied cell concentrations was added. The cell clusters were collected through centrifugation at 160 × *g* for 10 min and incubation for 24 h, and then transferred to an agarose-coated 96-well plate pre-added with culture medium. The cell clusters were cultured and grown for two more days to achieve MTSs of various sizes. A series of MTSs of various sizes (86.4, 128.9, 171.4, 215.8, 260.0, 370.0, and 449.5 μm in diameter) were made through varying the initial cell concentration.

### NIR-II SERS measurements

Raman spectroscopy was carried out on a Renishaw inVia Qontor confocal Raman microscope system equipped with an Andor InGaAs linear array detector and a ×5 (N.A. = 0.12) or ×50 (N.A. = 0.5) long working distance objective lens. A 1064 nm-Nd:YAG laser was employed in conjugation with holographic gratings of 830 lines/mm. All SERS spectra were processed and background-corrected using the Renishaw WiRE 5.3 software. To evaluate the NIR-II SERS performance of pc-AuAg NSs of various plasmon resonance wavelengths, pc-AuAg NSs of various concentrations were magnetically stirred with the resonant Raman reporter (IR-1061 dye, 1.0 μM) in aqueous suspensions. The mixture was then subjected to the SERS measurement with a 1064 nm laser at a power of 7.85 mW on the sample and integration time of 2 s, unless otherwise noted. Forty SERS spectra from different locations were averaged to represent the SERS signal for quantitative analysis. The analytical SERS enhancement factor (EF) was estimated by the following formula^27,38,39^:

1 $${{{{{\rm{EF}}}}}}=\frac{{I}_{{{{{{\rm{SERS}}}}}}}}{{I}_{{{{{{\rm{Raman}}}}}}}}\cdot \frac{{C}_{{{{{{\rm{Raman}}}}}}}}{{C}_{{{{{{\rm{SERS}}}}}}}}$$

where *I*_SERS_ and *I*_Raman_ represent the SERS intensity and spontaneous Raman intensity at 1557 cm^−1^ of IR-1061 dye, respectively; *C*_SERS_ and *C*_Raman_ are the concentrations of IR-1061 dye in the presence and absence of pc-AuAg NSs, respectively.

To conduct in vitro NIR-II SERS imaging of MTSs, 4T1 MTSs of various sizes were incubated in culture medium containing 50 μg/mL NIR-II SERS probes. After 12 h incubation, the MTSs were washed with fresh DMEM medium, which were then subjected to NIR-II SERS imaging measurements with a ×50 objective lens and a step size of 20 μm (laser power: 3.1 mW; integration time: 0.5 s). All SERS images were created by using a DCLS algorithm in the WiRE 5.3 software.

For in vivo studies of implanted MTSs, the 4T1 MTSs of different sizes obtained above were first incubated in DMEM medium containing 50 μg/mL NIR-II SERS probes for 12 h, and then washed with fresh DMEM medium. Afterward, the treated MTSs were implanted into a living mouse at a 2 mm depth beneath the skin, which was then subjected to the NIR-II SERS measurements using a ×5 objective lens at the laser power of 7.4 mW and integration time of 1 s.

For ex vivo and in vivo studies, subcutaneous 4T1 tumor xenograft models were established by inoculating 4 × 10^6^ 4T1 cells in 50 μL PBS into the right sub-dermal dorsal area of female Balb/c mice. Once the tumor grew to ~150 mm^3^, the mice were then used for animal experiments. Specifically, 200 μL of 2 mg/mL NIR-II SERS probes in PBS was intravenously administrated into 4T1 tumor-bearing mice (*n* = 3). The tumor was resected at 12 h post-administration from the treated mouse for the NIR-II SERS imaging measurement with the laser power of 25.6 mW at the sample and integration time of 2 s, and a step size of 750 μm. Furthermore, in vivo NIR-II SERS imaging of the tumor in living mice was carried out in 4T1 tumor-bearing mouse at 12 h post-administration under the same condition as that for the ex vivo study.

### Reporting summary

Further information on research design is available in the Nature Research Reporting Summary linked to this article.

## Supplementary information

Supplementary Information

Reporting Summary

## Data Availability

The data that support the findings of this study are available within the article, its Supplementary Information or from the corresponding author upon request. Source data are provided with this paper.

## Source data

Source Data
