# Supplementary material for: Near-infrared II plasmonic porous cubic nanoshells for in vivo noninvasive SERS visualization of sub-millimeter microtumors
Source: Nat Commun. 2022 Sep 6;13:5249. doi: 10.1038/s41467-022-32975-w (PMC9448796; doi:10.1038/s41467-022-32975-w)
Supplement: Supplementary file 1 — Supplementary Information [file 41467_2022_32975_MOESM1_ESM.pdf]

Supplementary Information

Near-infrared II plasmonic porous cubic nanoshells for in vivo noninvasive SERS visualization of sub-millimeter microtumors

Linhu Li[1], Renting Jiang[1], Beibei Shan[1], Yaxuan Lu[1], Chao Zheng[2], and Ming Li*[1]

[1]*School of Materials Science and Engineering, Central South University, Changsha, Hunan 410083, China*

[2]*Department of Breast Surgery, The Second Hospital, Cheeloo College of Medicine, Shandong University, Jinan, Shandong, 250033, China*

*Correspondence and requests for materials should be addressed to M.L. (E-mail: liming0823@csu.edu.cn or liming0823@gmail.com

Supplementary Methods

Chemicals and materials. All chemicals were used as received without any further purification. Ethylene glycol (EG, $C_2H_6O_2$, >99.0%), silver trifluoroacetate (CF_3COOAg, 98.0%), hexadecyltrimethylammonium chloride (CTAC, $CH_3(CH_2)_{15}N(Cl)(CH_3)_3$, 97.0%), and sodium sulfide ($Na_2S \cdot 9H_2O$, 99.99%, metals basis) were ordered from Aladdin Chemistry Co., Ltd. Poly(vinyl pyrrolidone) (PVP, $(C_6H_9NO)_n$, average molecular weight ≈ 29 kg/mol), poly(sodium 4-styrenesulfonate) (PSS), and IR-1061 dye (80.0%) were purchased from Sigma-Aldrich Shanghai Trading Co., Ltd. Polyethyleneimine (PEI, > 99%, average molecular weight ≈ 10 kg/mol) was purchased from Adamas Reagent, Ltd. Hyaluronic acid (HA, $(C_{14}H_{21}NO_{11})_n$, average molecular weight ≈ 10 kg/mol) was obtained from Dalian Meilunbio. Acetone (reagent grade), L-ascorbic acid (H_2Asc, $C_6H_8O_6$, AR), hydrochloric acid (HCl, 36.5-38.0%), and hydrogen peroxide solution (H_2O_2, 30 vol%, AR) were purchased from Sinopharm Chemical Reagent Co., Ltd. Sodium hydroxide (NaOH, >96.0%, AR) was produced with Shantou Xilong Scientific Co., Ltd. Chloroauric acid ($HAuCl_4 \cdot 4H_2O$, 99.0% trace metals basis) was provided by Shanghai Civi Chemical Technology Co., Ltd. Ultrapure water (resistivity: 18.2 MΩ·cm at 25 °C) produced with a Millipore Direct-Q3 UV system was used throughout the experiments. All glassware and stir bars were cleaned with aqua regia prior to use, followed by being thoroughly rinsed with ultrapure water (**CAUTION**: aqua regia is strongly corrosive and dangerous, and should be handled with extreme care!).

Characterization. Optical extinction spectra were collected on an Agilent Cary 5000 UV-vis-NIR spectrophotometer. Transmission electron microscopy (TEM) images were captured on a FEI Tecnai G^2 F20 S-TWIN TMP transmission electron microscope operating at 200 kV. High angle annular dark field-scanning transmission electron microscopy (HAADF-STEM) imaging and energy-dispersive X-ray spectroscopy (EDS) elemental mapping were performed using a Thermo Fisher Scientific Talos™ F200X scanning/transmission electron microscope microscope at 200 kV. The samples were prepared by dropping 1.5 μL of aqueous suspension of samples on a 300-mesh copper grid with carbon film and then being dried in the air. Size distribution of samples was statistically determined from the TEM data using

the ImageJ analysis software with more than 200 particles counted for each sample. Scanning electron microscopy (SEM) images were measured on a Hitachi Regulus 8230 field-emission scanning electron microscope at 15 kV. Quantitative composition analysis of Au and Ag was performed using inductively coupled plasma - optical emission spectrometry (ICP-OES) on an Agilent 5100 inductively coupled plasma - optical emission spectrometer or inductively coupled plasma - mass spectrometry (ICP-MS) using a PerkinElmer NEXION 2000 inductively coupled plasma - mass spectrometer. The samples were prepared by dissolving the samples in aqua regia and then being diluted with ultrapure water. Au and Ag standard solutions were diluted with ultrapure water to achieve a series of concentrations for creating the calibration curve of the ICP-OES or ICP-MS measurements. Zeta potential and hydrodynamic size measurements were carried out on a Malvern Zetasizer Nano ZSE ZEN3700 instrument. The fluorescence images were captured with a Zeiss Axio Vert. A1 inverted fluorescence microscope.

Synthesis of Ag NCs. Ag NCs were synthesized according to a polyol process previously developed by Xia's group[1]. In a typical synthesis, a vial containing 5 mL EG was placed in an oil bath maintained at 150 °C. After being heated for 20 min, 60 μL of an EG solution with 0.72 mg/mL Na_2S was added under magnetic stirring. Afterwards, 0.5 mL of 3 mM HCl in EG was rapidly injected into the reaction solution, followed by the introduction of 1.25 mL EG containing 20 mg/mL PVP. After 2 min, 0.4 mL of 282 mM CF_3COOAg in EG was titrated into the mixtures. During the entire experiments, the cap was kept on the top of the vial. Following the complete addition of CF_3COOAg, the reaction was terminated at 45 min by placing the vial in an ice-water bath. The products were washed three times successively with acetone and ultrapure water, which was then dispersed in ultrapure water for further use. The concentration of the as-synthesized Ag NCs was estimated to be 0.74 mg/mL by ICP-OES.

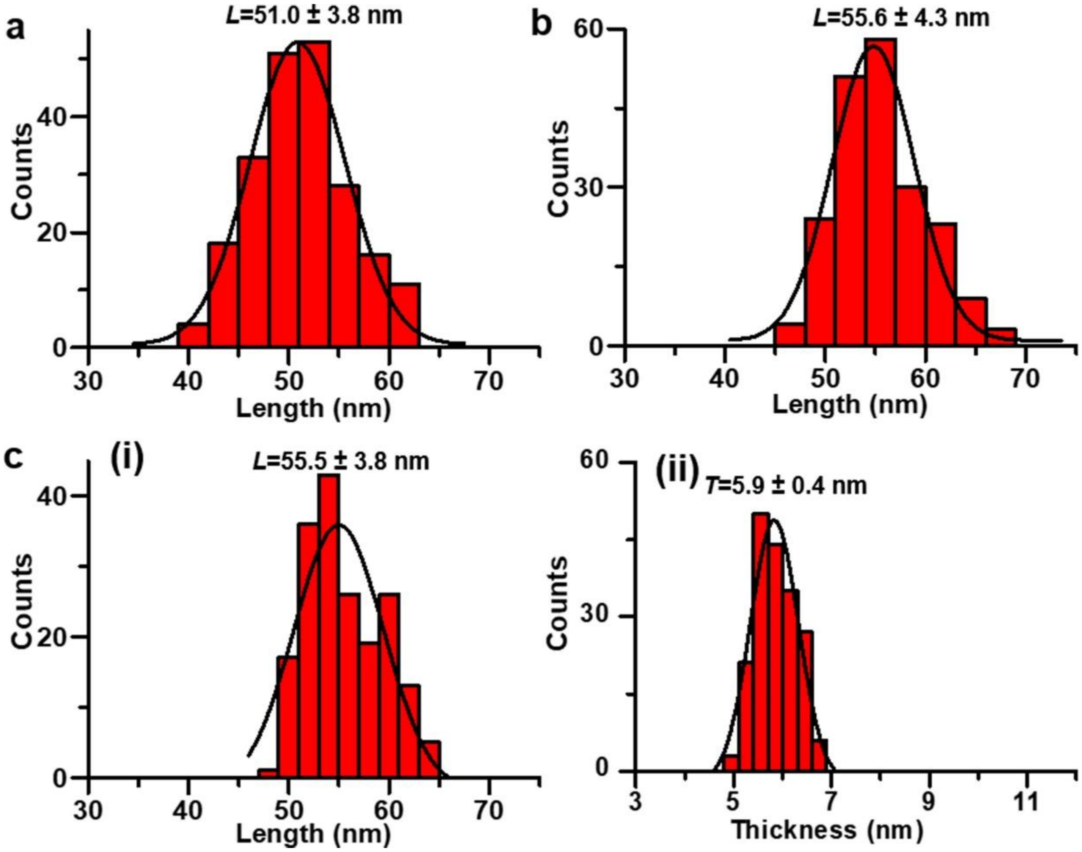

Supplementary Fig. 1. Edge length distribution of (a) Ag NCs, (b) Ag@Au NCs, and (c) (i) edge length and (ii) thickness distributions of the corresponding pc-AuAg NSs after H_2O_2 etching. Ag@Au NCs used here was synthesized with 0.4 mL of 1.0 mM $HAuCl_4$. The pc-AuAg NSs have a similar edge length to the original Ag@Au NCs.

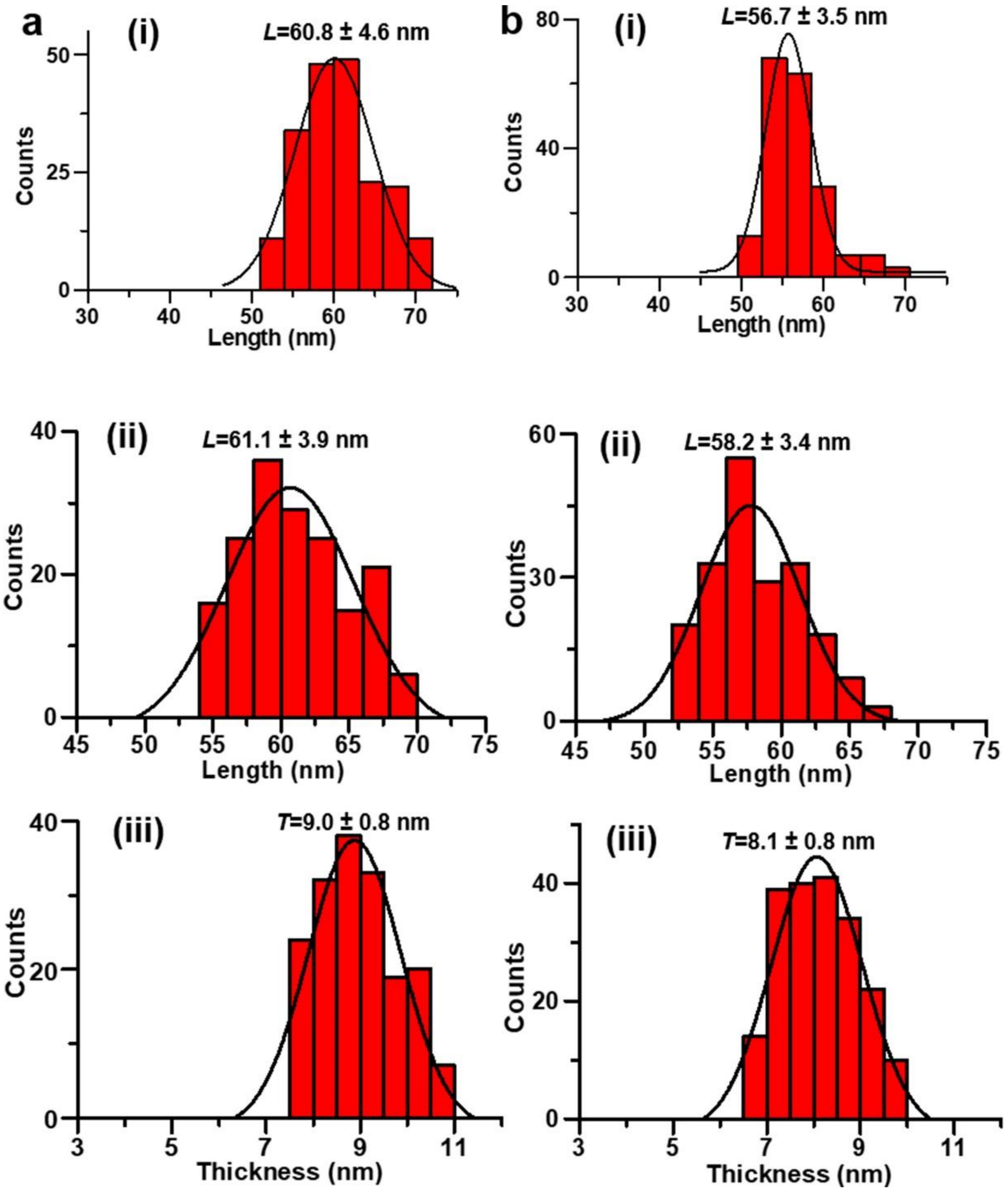

Supplementary Fig. 2. (i) Edge length distribution of Ag@Au NCs, (ii) edge length distribution and (iii) shell thickness distribution of the corresponding pc-AuAg NSs synthesized with (a) 1.2 mL and (b) 0.6 mL of 1.0 mM $HAuCl_4$.

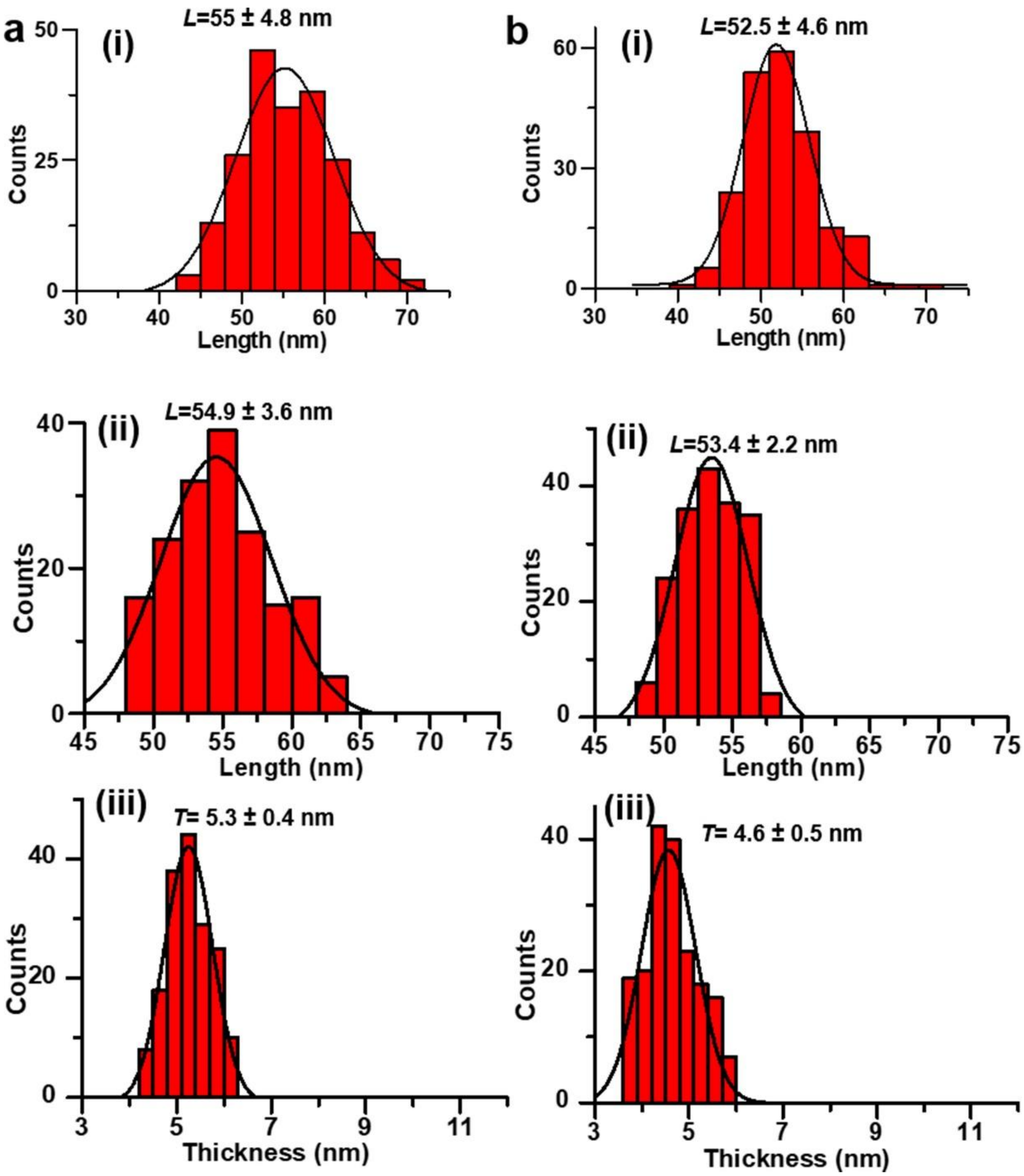

Supplementary Fig. 3. (i) Edge length distribution of Ag@Au NCs, (ii) edge length distribution and (iii) shell thickness distribution of the corresponding pc-AuAg NSs synthesized with (a) 0.3 mL and (b) 0.1 mL of 1.0 mM $HAuCl_4$.

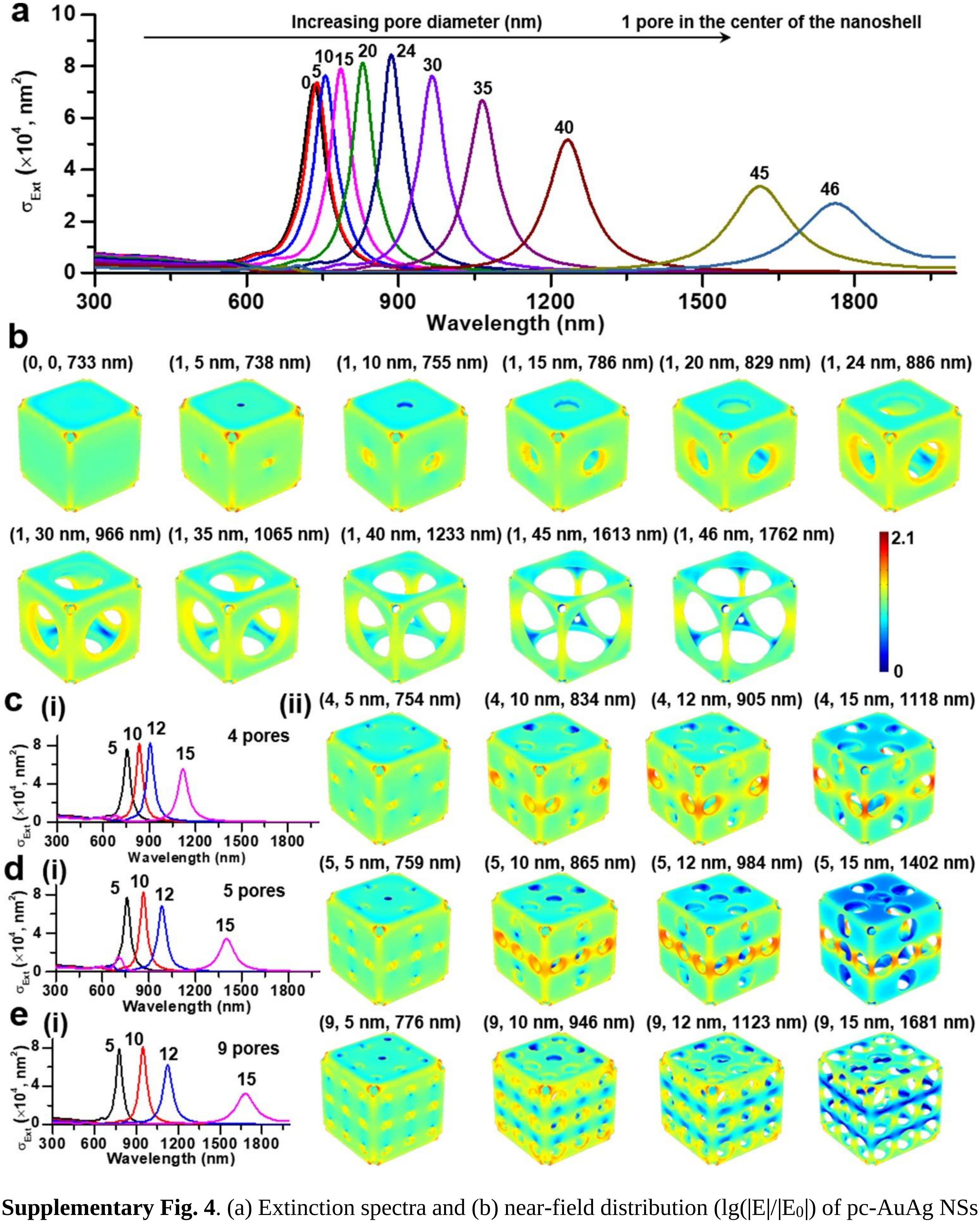

Supplementary Fig. 4. (a) Extinction spectra and (b) near-field distribution (lg(|E|/|E_0|) of pc-AuAg NSs containing one pore in the center of each side face of the nanoshell with varied pore diameters. (c-e) (i)

Extinction spectra and (ii) near-field distribution ($lg(|E|/|E_0|)$) of pc-AuAg NSs containing (c) four pores (d) five pores and (e) nine pores with varied pore diameters. (c-e) share the same color bars as b.

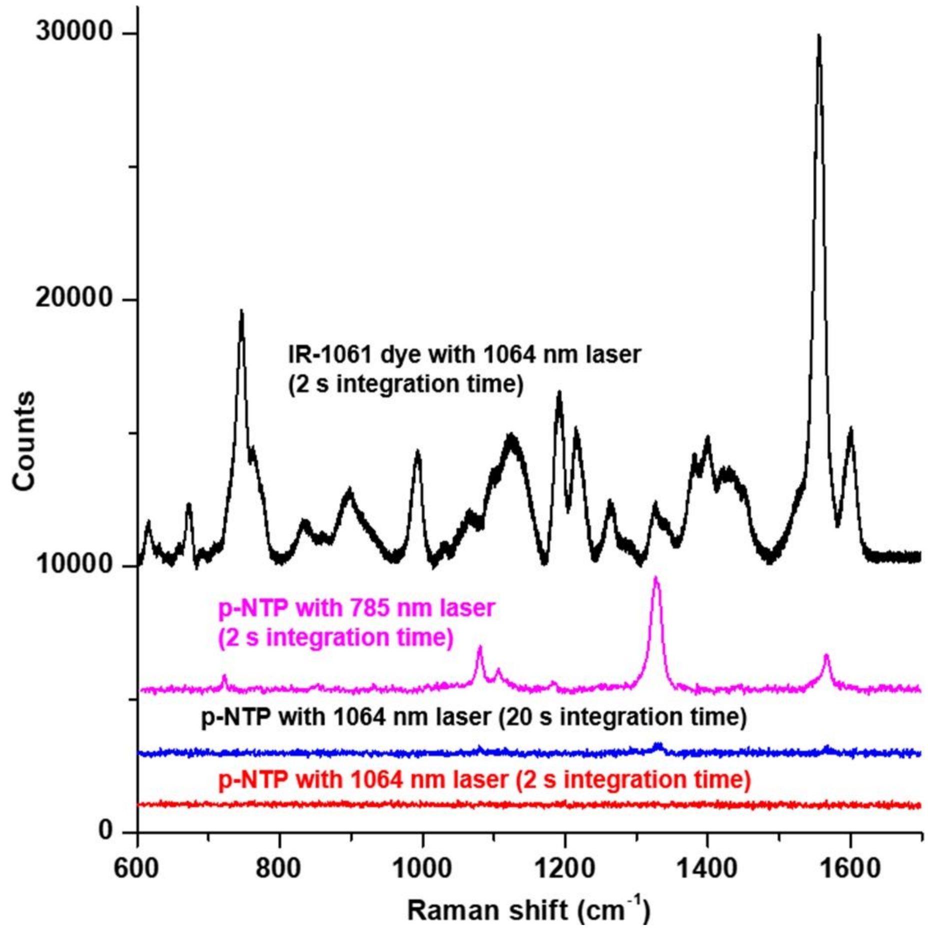

Supplementary Fig. 5. SERS spectra of SERS probes made with pc-AuAg NSs-945 nm encoded respectively with resonant Raman molecule IR-1061 dye and non-resonant Raman molecule p-NTP, subjected to SERS measurements under 1064 nm laser or 785 nm laser. The laser powers of the 1064 nm laser and 785 nm laser are 7.85 mW and 6.56 mW, respectively.

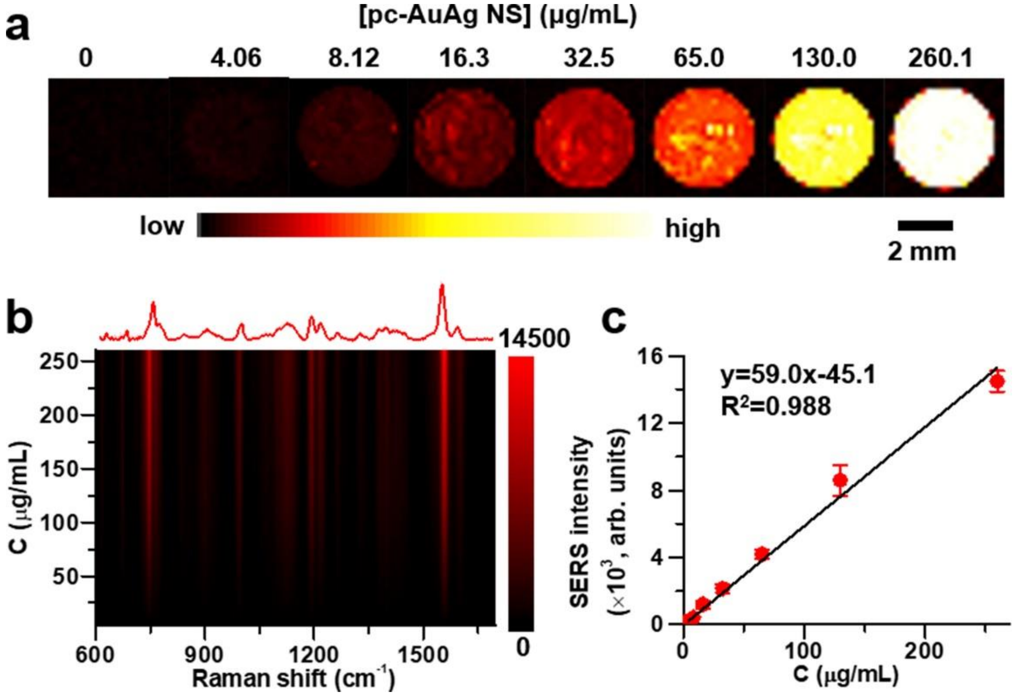

Supplementary Fig. 6. (a) SERS images created with the peak intensity at 1557 cm^{-1} and (b) concentration-dependent dependent SERS spectra of agarose phantoms containing of various concentrations of pc-AuAg NSs-945 nm and 1.0 μM IR-1061 dye. (c) Change of the SERS intensity at 1557 cm^{-1} with the concentration of pc-AuAg NSs-945 nm. Data are presented as mean ± SD (n=5 independent experiments).

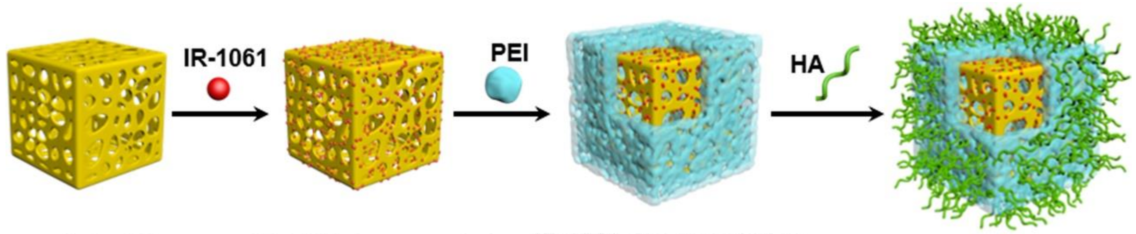

Supplementary Fig. 7. Schematic illustration of the preparation process of the NIR-II SERS probe. The NIR-II plasmonic pc-AuAg NS was first encoded with IR-1061 dye, followed by the PEI coating layer and HA targeting modification.

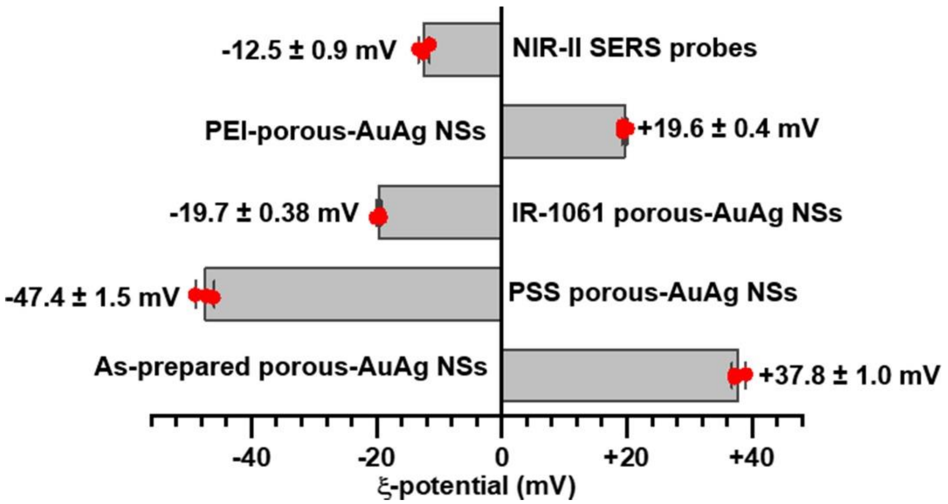

Supplementary Fig. 8. Zeta potential of pc-AuAg NSs after various treatments during the preparation of NIR-II SERS probes. Data are presented as mean ± SD (n=3 independent experiments), and individual data points are shown as red dots.

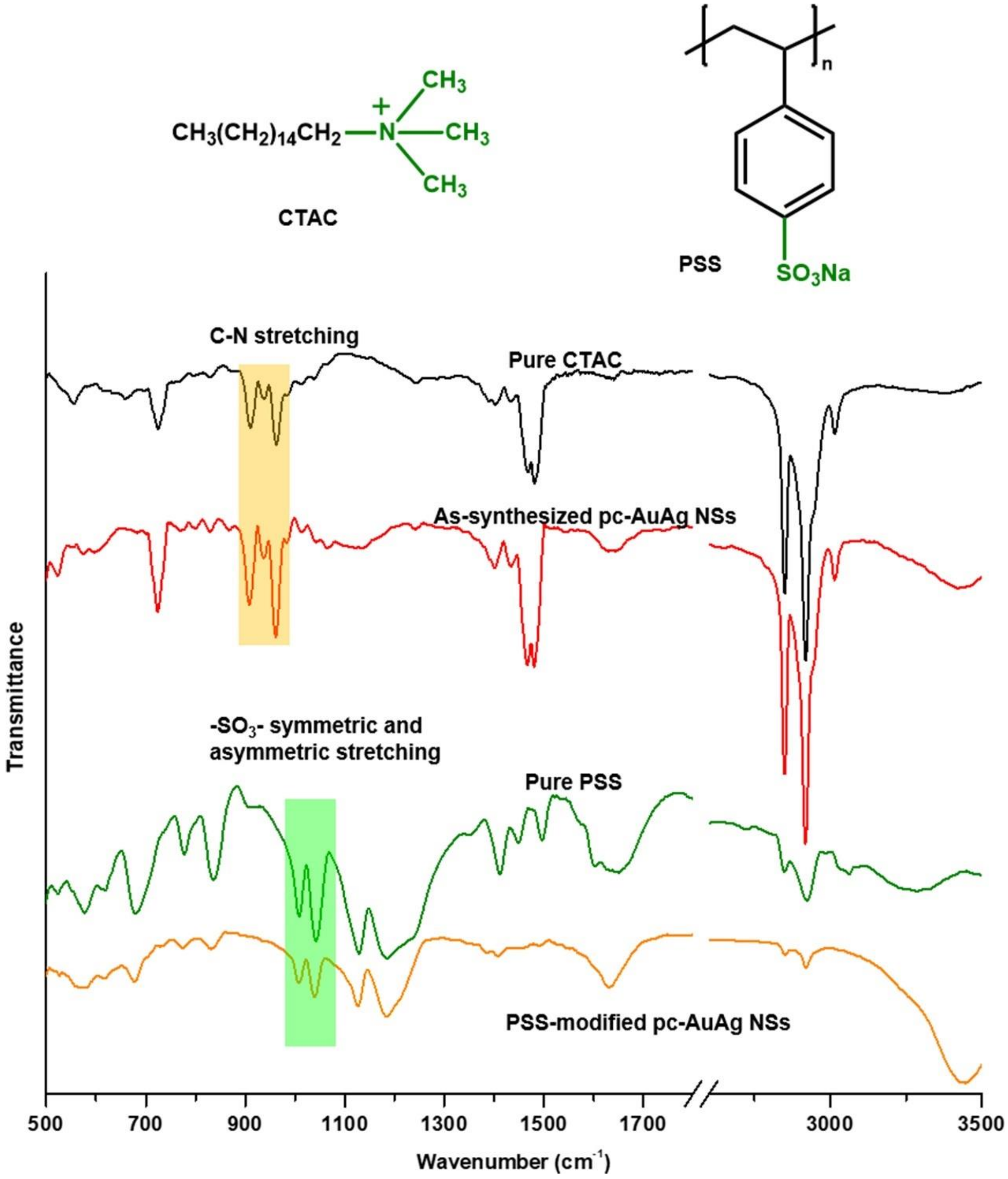

Supplementary Fig. 9. FTIR spectra of pure CTAC, pure PSS, as-synthesized pc-AuAg NSs, and PSS-modified pc-AuAg NSs, respectively. The FTIR bands characteristic of -C-N- at 920-950 cm^{-1}, indicating the presence of CTAC in the as-synthesized pc-AuAg NSs, while disappearance of the -C-N- bands associated with the show-up of characteristic bands of $-SO_3-$ at 1010 and 1040 cm^{-1} justify the complete replacement of CTAC by PSS on the pc-AuAg NSs after treatment in the solution PSS.

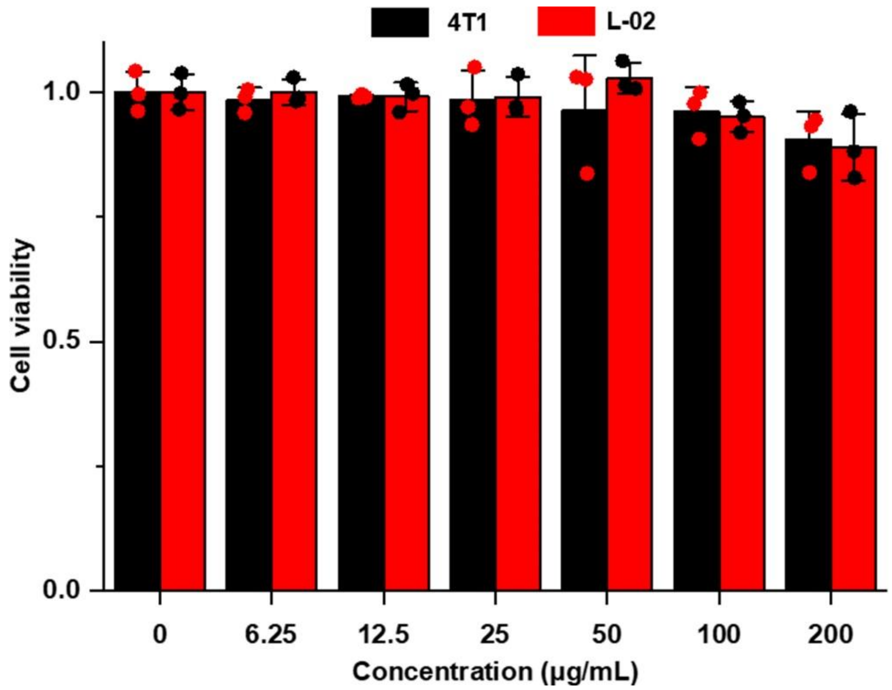

Supplementary Fig. 10. In vitro cell viability assay of 4T1 cells and L-02 cells after treatments with NIR-II SERS probes at varied concentrations for 24 h. Data are presented as mean ± SD (n=3 independent experiments), and individual data points are shown as red dots for 4T1 cells and black dots for L-02 cells.

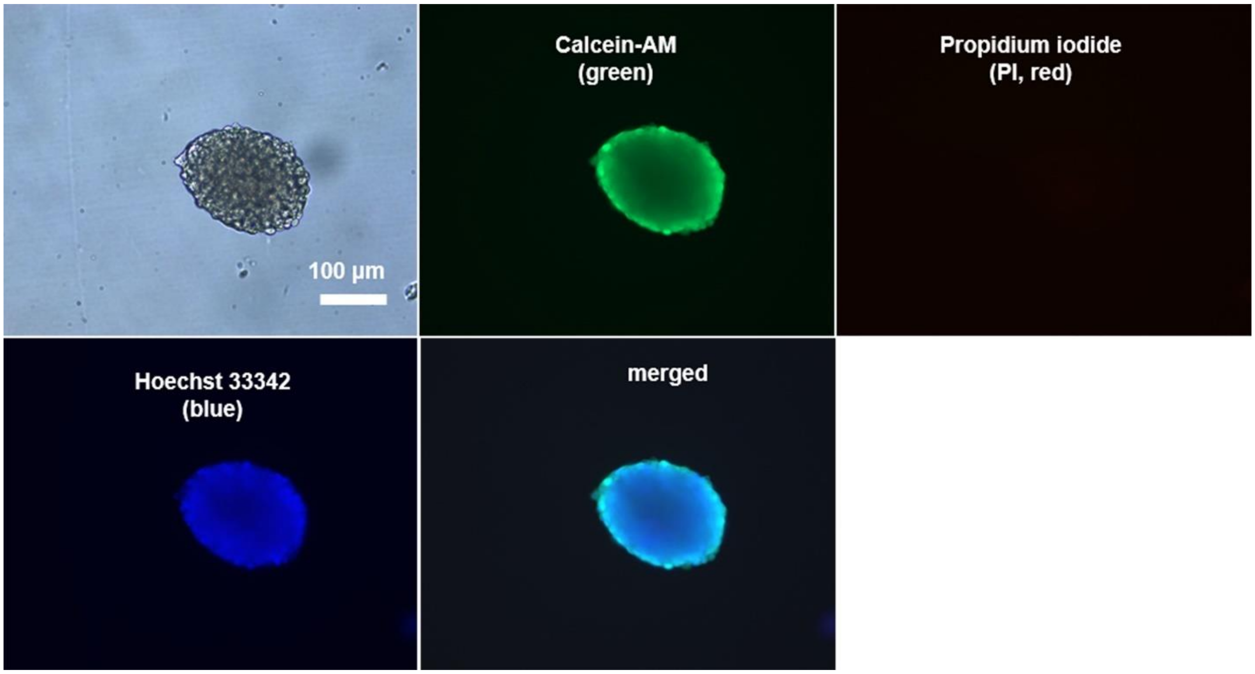

Supplementary Fig. 11. Optical photographs and fluorescence images of a representative MTS co-stained with Calcein-AM (green), Hoechst 33342 (blue) and propidium iodide (PI, red), confirming live cells in the MTS and negligible dead cells at the center of the MTS. Scale bar: 100 μm.

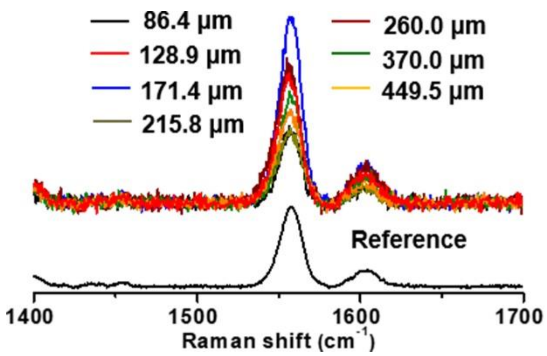

Supplementary Fig. 12. Representative SERS spectra of multicellular tumor spheroids with various diameters (86.4, 128.9, 171.4, 215.8, 260.0, 370.0, and 449.5 μm), corresponding to the SERS images as shown in Figure 5E. The SERS spectrum of NIR-II SERS probes with the 1064 nm laser excitation was shown as the reference for the creation of SERS images.

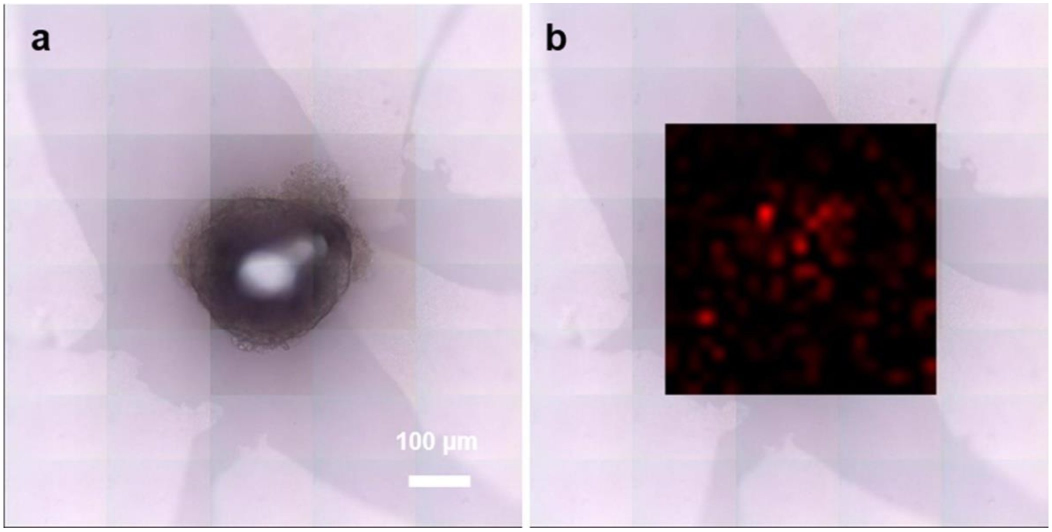

Supplementary Fig. 13. (a) Optical photograph and (b) overlayered SERS image of the MTS incubated with mPEG-modified NIR-II probes, showing weak SERS signal in the MTS.

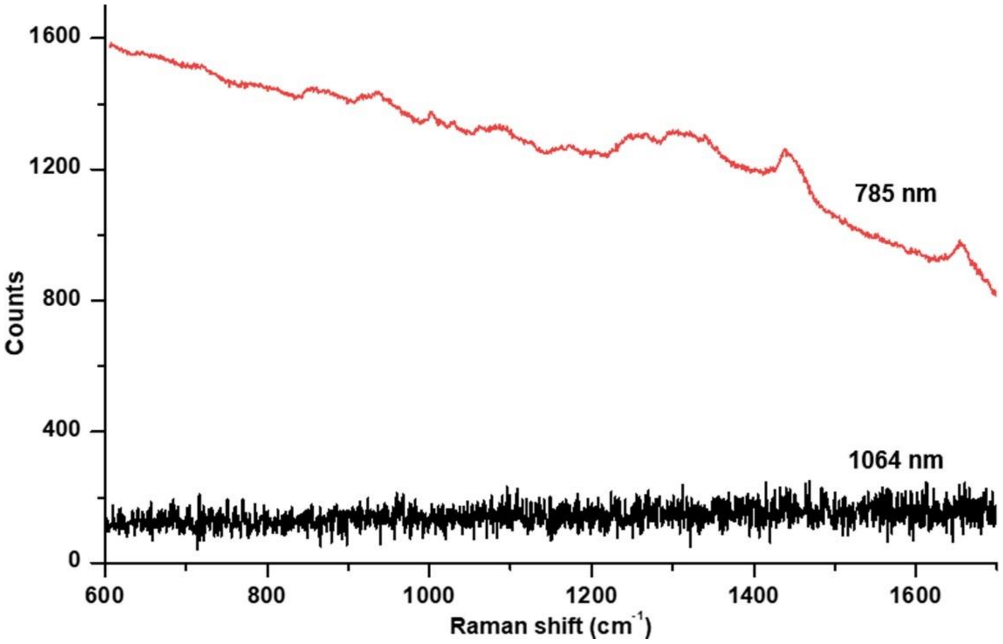

Supplementary Fig. 14. Average Raman spectra from 441 measurements of the skin directly obtained from a living healthy mouse using 785 nm and 1064 nm lasers, respectively. The spontaneous Raman measurements were performed with 5× objective lens, 23.9 mW laser power, and 2.0 s integration time for 785 nm laser, and with 5× objective lens, 25.6 mW laser power, and 2.0 s integration time for 1064 nm laser. It clearly shows the strong background signal and obvious intrinsic Raman bands under 785 nm laser, while flat baseline and weak background interference can be seen under 1064 nm laser.

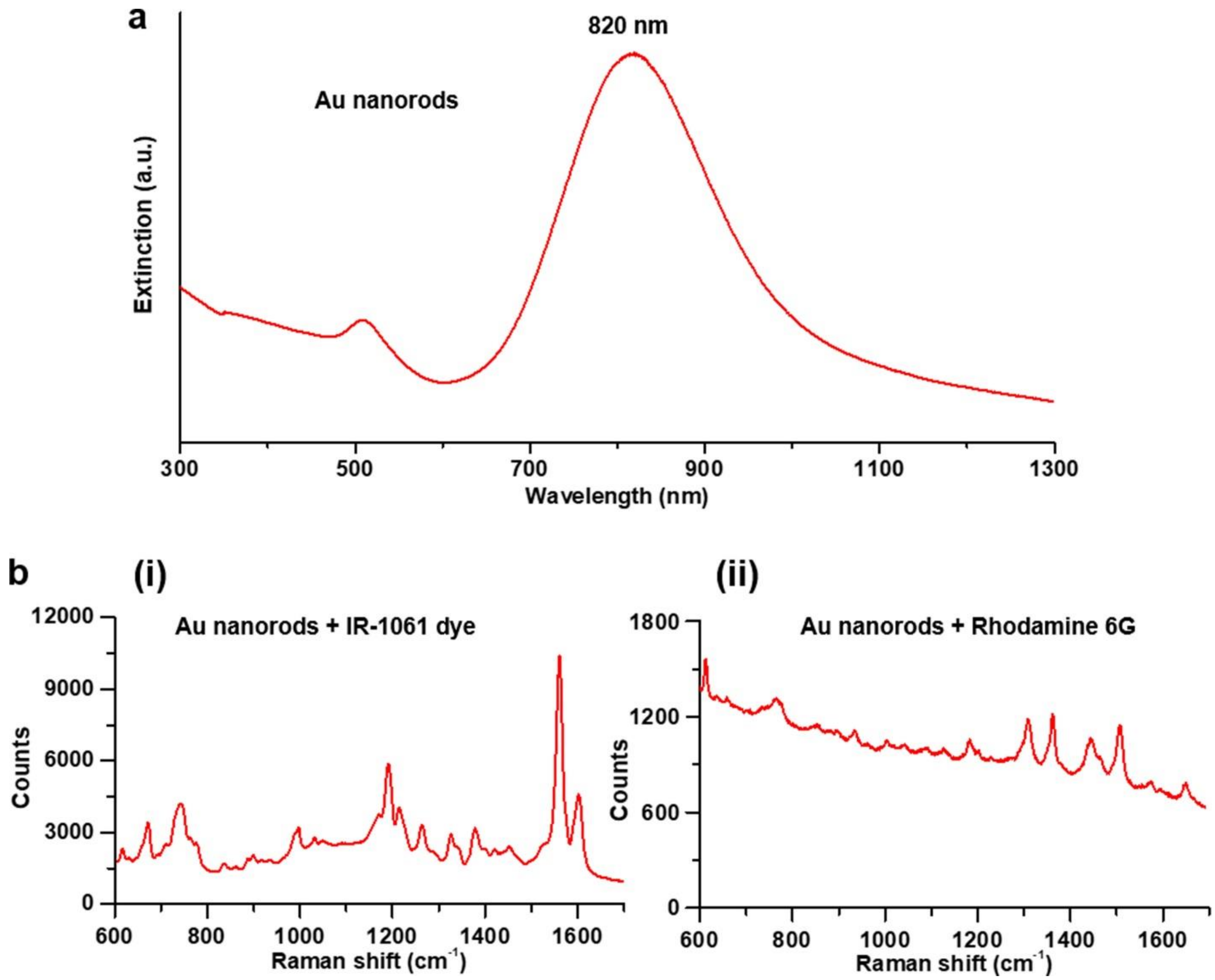

Supplementary Fig. 15. (a) Optical extinction spectrum of Au nanorods in water, showing a longitudinal plasmon resonance wavelength of 820 nm, and (b) SERS spectra of (i) IR-1061 dye and (ii) Rhodamine 6G both at 1.0 μM in an aqueous solution of 200 μg/mL Au nanorods under 785 nm laser. The SERS measurements were conducted with 50× objective lens, 6.6 mW laser power, and 2 s integration time.

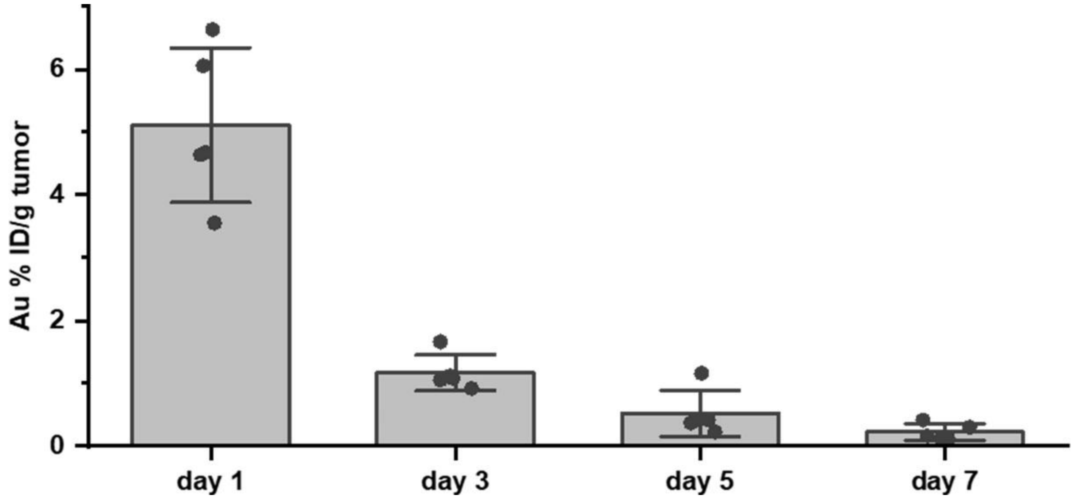

Supplementary Fig. 16. The content of the NIR-II SERS probes in the tumor of 4T1 tumor-bearing mice after intravenous administration with NIR-II SERS probes, quantified by ICP-MS based on the Au mass percentage. Data are presented as mean ± SD (n=5 independent experiments, and individual data points are shown as black dots).

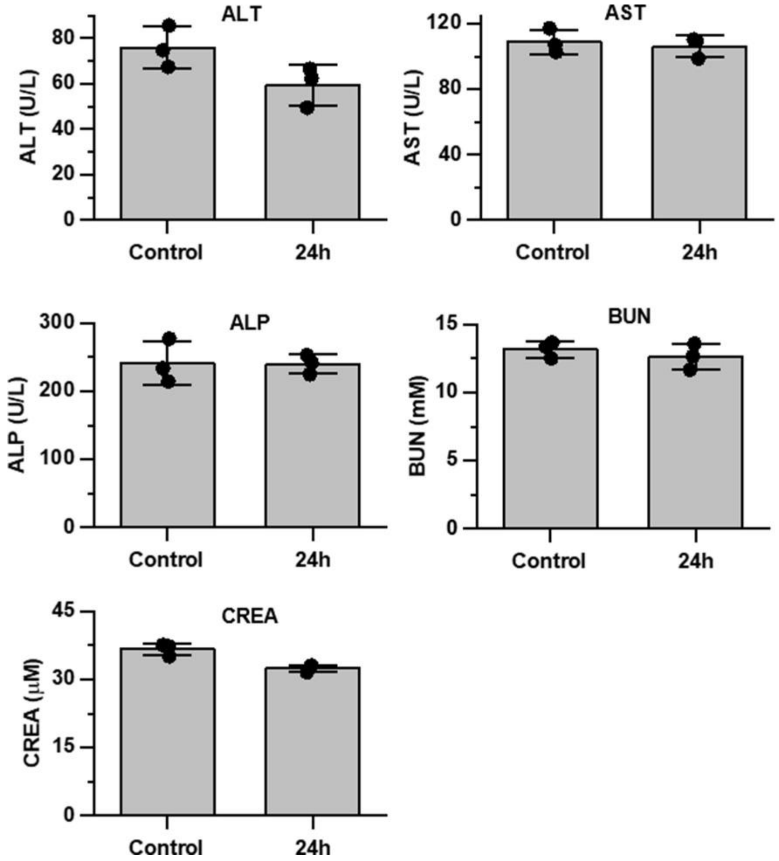

Supplementary Fig. 17. Blood biochemical levels of healthy mice at 24 h after intravenous administration with NIR-II SERS probes (200 μL, 20 mg/kg) (n=3). PBS (200 μL) was used as the control. No obvious difference was observed, indicating no systemic toxicity of the present NIR-II SERS probes. Data are presented as mean ± SD (n=3 independent experiments, and individual data points are shown as black dots).

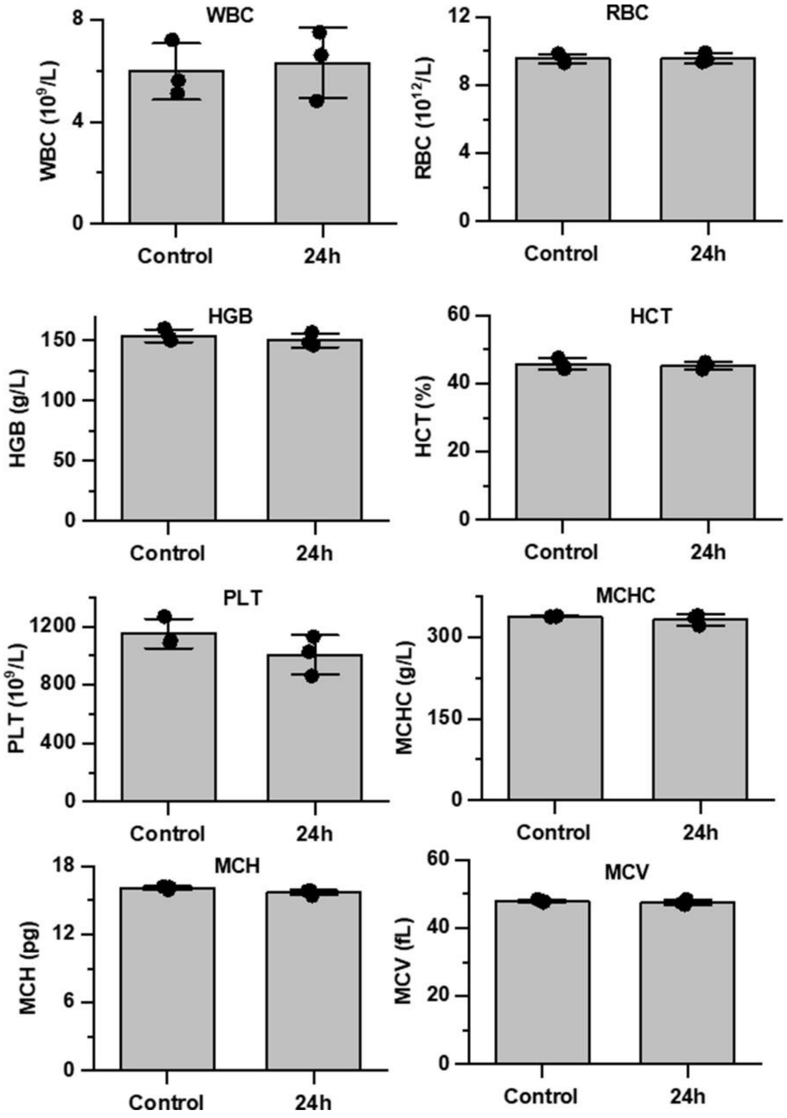

Supplementary Fig. 18. Hematological analysis of healthy mice at 24 h after intravenous administration with NIR-II SERS probes (200 μL, 20 mg/kg) (n=3). PBS (200 μL) was used as the control. No obvious difference was observed, indicating no systemic toxicity, no liver/kidney dysfunction and no obvious adverse effects on immune response of the present NIR-II SERS probes. Data are presented as mean ± SD (n=3 independent experiments, and individual data points are shown as black dots).

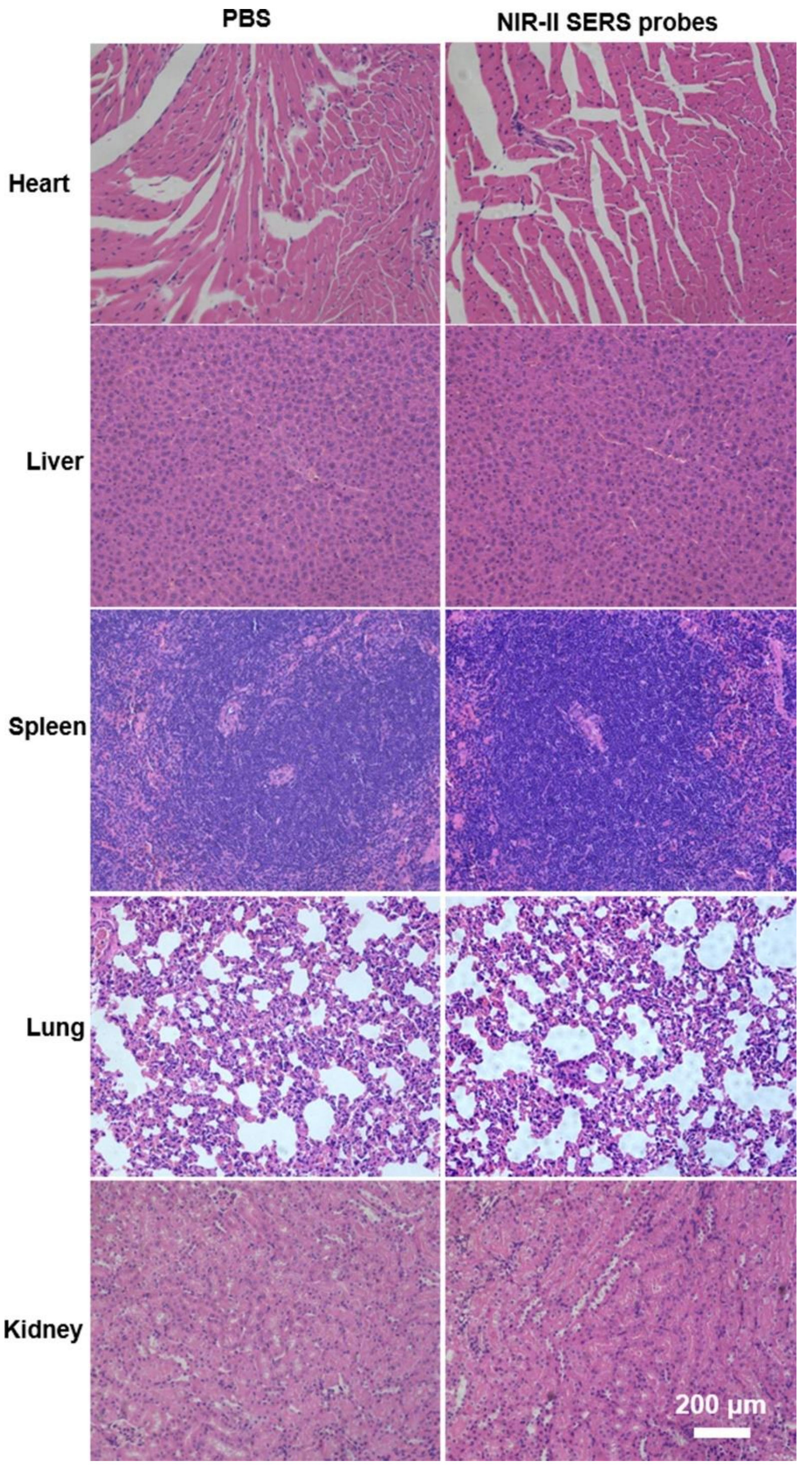

Supplementary Fig. 19. Hematoxylin-eosin (H&E) stained images of tissue slices of major organs (heart, liver, spleen, lung, and kidney) from 4T1 tumor-bearing mice at day 14 after treatment with PBS and NIR-II SERS probes, respectively.

Supplementary references

1. Skrabalak, S.E., Au, L., Li, X. & Xia, Y. Facile synthesis of Ag nanocubes and Au nanocages. *Nat. Protoc*. **2**(9), 2182-2190 (2007).
