# Supplementary material for: Near-infrared II plasmonic porous cubic nanoshells for in vivo noninvasive SERS visualization of sub-millimeter microtumors
Source: Nat Commun. 2022 Sep 6;13:5249. doi: 10.1038/s41467-022-32975-w (PMC9448796; doi:10.1038/s41467-022-32975-w)
Supplement: Supplementary file 2 — Reporting Summary [file 41467_2022_32975_MOESM2_ESM.pdf]

nature portfolio

Corresponding author(s): Ming Li

Last updated by author(s): Aug 25, 2022

Reporting Summary

Nature Portfolio wishes to improve the reproducibility of the work that we publish. This form provides structure for consistency and transparency in reporting. For further information on Nature Portfolio policies, see our Editorial Policies and the Editorial Policy Checklist.

Statistics

For all statistical analyses, confirm that the following items are present in the figure legend, table legend, main text, or Methods section.

n/a	Confirmed	
☐	☒	The exact sample size (*n*) for each experimental group/condition, given as a discrete number and unit of measurement
☐	☒	A statement on whether measurements were taken from distinct samples or whether the same sample was measured repeatedly
☒	☐	The statistical test(s) used AND whether they are one- or two-sided<br>*Only common tests should be described solely by name; describe more complex techniques in the Methods section.*
☒	☐	A description of all covariates tested
☒	☐	A description of any assumptions or corrections, such as tests of normality and adjustment for multiple comparisons
☐	☒	A full description of the statistical parameters including central tendency (e.g. means) or other basic estimates (e.g. regression coefficient) AND variation (e.g. standard deviation) or associated estimates of uncertainty (e.g. confidence intervals)
☒	☐	For null hypothesis testing, the test statistic (e.g. *F*, *t*, *r*) with confidence intervals, effect sizes, degrees of freedom and *P* value noted<br>*Give P values as exact values whenever suitable.*
☒	☐	For Bayesian analysis, information on the choice of priors and Markov chain Monte Carlo settings
☒	☐	For hierarchical and complex designs, identification of the appropriate level for tests and full reporting of outcomes
☒	☐	Estimates of effect sizes (e.g. Cohen's *d*, Pearson's *r*), indicating how they were calculated

Our web collection on statistics for biologists contains articles on many of the points above.

Software and code

Policy information about availability of computer code

Data collection	The calculations of optical extinction spectra and three-dimensional (3D) near-field distribution were performed by finite element method using a commercial software package COMSOL Multiphysics 5.5. Optical extinction spectra were collected on an Agilent Cary 5000 UV-vis-NIR spectrophotometer. Transmission electron microscopy (TEM) images were captured on a FEI Tecnai G2 F20 S-TWIN TMP transmission electron microscope operating at 200 kV. High angle annular dark field-scanning transmission electron microscopy (HAADF-STEM) imaging and energy-dispersive X-ray spectroscopy (EDS) elemental mapping were performed using a Thermo Fisher Scientific TalosTM F200X scanning/transmission electron microscope microscope at 200 kV. The samples were prepared by dropping 1.5 μL of aqueous suspension of samples on a 300-mesh copper grid with carbon film and then being dried in the air. Size distribution of samples was statistically determined from the TEM data using the ImageJ analysis software with more than 200 particles counted for each sample. Scanning electron microscopy (SEM) images were measured on a Hitachi Regulus 8230 field-emission scanning electron microscope at 15 kV. Quantitative composition analysis of Au and Ag was performed using inductively coupled plasma - optical emission spectrometry (ICP-OES) on an Agilent 5100 inductively coupled plasma - optical emission spectrometer or inductively coupled plasma - mass spectrometry (ICP-MS) using a PerkinElmer NEXION 2000 inductively coupled plasma - mass spectrometer. The samples were prepared by dissolving the samples in aqua regia and then being diluted with ultrapure water. Au and Ag standard solutions were diluted with ultrapure water to achieve a series of concentrations for creating the calibration curve of the ICP-OES or ICP-MS measurements. Zeta potential and hydrodynamic size measurements were carried out on a Malvern Zetasizer Nano ZSE ZEN3700 instrument. The fluorescence images were captured with a Zeiss Axio Vert. A1 inverted fluorescence microscope. Raman spectroscopy was carried out on a Renishaw inVia Qontor confocal Raman microscope system
Data analysis	Origin 8.5/Origin 2019/COMSOL Multiphysics 5.5/ImageJ 1.49m/DCLS algorithm in the WiRE 5.3 software

For manuscripts utilizing custom algorithms or software that are central to the research but not yet described in published literature, software must be made available to editors and reviewers. We strongly encourage code deposition in a community repository (e.g. GitHub). See the Nature Portfolio guidelines for submitting code & software for further information.

Data

Policy information about availability of data

All manuscripts must include a data availability statement. This statement should provide the following information, where applicable:

- Accession codes, unique identifiers, or web links for publicly available datasets
- A description of any restrictions on data availability
- For clinical datasets or third party data, please ensure that the statement adheres to our policy

The data that support the findings of this study are available within the article, its Supplementary Information or from the corresponding author upon request. Source Data are provided with this article.

Field-specific reporting

Please select the one below that is the best fit for your research. If you are not sure, read the appropriate sections before making your selection.

☒ Life sciences ☐ Behavioural & social sciences ☐ Ecological, evolutionary & environmental sciences

For a reference copy of the document with all sections, see nature.com/documents/nr-reporting-summary-flat.pdf

Life sciences study design

All studies must disclose on these points even when the disclosure is negative.

Sample size	The SERS enhancement factor was calculated based on n=37 independent experiments. The size of all nanoparticles was statistically estimated based on more than 150 nanoparticles from the TEM data. Three or five independent experiments were performed for SERS measurements, zeta potential measurement, and in vitro and in vivo biological analysis throughout this work.
Data exclusions	No data was excluded.
Replication	All biological analysis was conducted in three or five independent experiments. results were presented as mean ± standard deviation.
Randomization	The allocations of fluorescence images, H&E image analysis and TEM/SEM images used this study were randomized by performing analysis at random positions.
Blinding	Characterizations of materials (DLS, TEM, SEM, fluorescence imaging, and SERS measurements) do not routinely use blinded samples since negligible effects from investigators take place. The cell viability assay and in vivo imaging experiments were blinded as the samples were labeled and analyzed by at least three different investigators.

Reporting for specific materials, systems and methods

We require information from authors about some types of materials, experimental systems and methods used in many studies. Here, indicate whether each material, system or method listed is relevant to your study. If you are not sure if a list item applies to your research, read the appropriate section before selecting a response.

Materials & experimental systems

n/a	Involved in the study	
☒	☐	Antibodies
☐	☒	Eukaryotic cell lines
☒	☐	Palaeontology and archaeology
☐	☒	Animals and other organisms
☒	☐	Human research participants
☒	☐	Clinical data
☒	☐	Dual use research of concern

Methods

n/a	Involved in the study	
☒	☐	ChIP-seq
☒	☐	Flow cytometry
☒	☐	MRI-based neuroimaging

Eukaryotic cell lines

Policy information about cell lines

Cell line source(s)	4T1 and L-02 cells were purchased from ATCC.
Authentication	The 4T1 and L-02 cell lines were not authenticated by the investigators but certified by the vendor (Shanghai Aoyinbio).
Mycoplasma contamination	No mycoplasma contamination was found for cell lines used in this study.
Commonly misidentified lines (See ICLAC register)	Both 4T1 and L-02 cells were certified by the vendor and then used in this study.

Animals and other organisms

Policy information about studies involving animals; ARRIVE guidelines recommended for reporting animal research

Laboratory animals	Female Balb/c mice (four weeks, 13-16 g) were ordered from Changzhou Cavens Laboratory Animal Co. Ltd. All mice were housed in the university animal facility under constant standard conditions (25 oC,40-70% humidity, and a 12 light-dark cycle).
Wild animals	No wild animals were used in this study.
Field-collected samples	No field-collected samples were used in this study.
Ethics oversight	All procedures of animal experiments were approved by the Institutional Animal Care and Use Committee at Central South University (Protocol No. 2020sydw0724).

Note that full information on the approval of the study protocol must also be provided in the manuscript.
